# UNC93B1 interacts with the calcium sensor STIM1 for efficient antigen cross-presentation in dendritic cells

**DOI:** 10.1038/s41467-017-01601-5

**Published:** 2017-11-21

**Authors:** Sophia Maschalidi, Paula Nunes-Hasler, Clarissa R Nascimento, Ignacio Sallent, Valérie Lannoy, Meriem Garfa-Traore, Nicolas Cagnard, Fernando E. Sepulveda, Pablo Vargas, Ana-Maria Lennon-Duménil, Peter van Endert, Thierry Capiod, Nicolas Demaurex, Guillaume Darrasse-Jèze, Bénédicte Manoury

**Affiliations:** 1 INSERM UMR1163, Laboratory of Normal and Pathological Homeostasis of the Immune System, Imagine Institute, 75015 Paris, France; 2 Faculté de médecine Paris Descartes, Université Paris Descartes, 75015 Paris, France; 3 Department of Cell Physiology and Metabolism, University of Geneva, CH-1211 Geneva, Switzerland; 4 Institut National de la Santé et de la Recherche Médicale, Unité 1151, 75015 Paris, France; 5 Centre National de la Recherche Scientifique, UMR 8253, 75015 Paris, France; 6 Cell Imaging and Bioinformatic Platform, INSERM US24 Structure Federative de Recherche Necker, 75015 Paris, France; 7 Institut Curie, PSL Research University, Centre National de la Recherche Scientifique, UMR 144, 75005 Paris, France; 8 Institut Pierre-Gilles de Genes, PSL Research University, 75005 Paris, France; 9 Institut National de la Santé et de la Recherché Médicale, Unité 932, Institut Curie, PSL Research University, 75005 Paris, France

## Abstract

Dendritic cells (DC) have the unique ability to present exogenous antigens via the major histocompatibility complex class I pathway to stimulate naive CD8^+^ T cells. In DCs with a non-functional mutation in *Unc93b1* (3d mutation), endosomal acidification, phagosomal maturation, antigen degradation, antigen export to the cytosol and the function of the store-operated-Ca^2+^-entry regulator STIM1 are impaired. These defects result in compromised antigen cross-presentation and anti-tumor responses in 3d-mutated mice. Here, we show that UNC93B1 interacts with the calcium sensor STIM1 in the endoplasmic reticulum, a critical step for STIM1 oligomerization and activation. Expression of a constitutively active STIM1 mutant, which no longer binds UNC93B1, restores antigen degradation and cross-presentation in 3d-mutated DCs. Furthermore, ablation of STIM1 in mouse and human cells leads to a decrease in cross-presentation. Our data indicate that the UNC93B1 and STIM1 cooperation is important for calcium flux and antigen cross-presentation in DCs.

## Abstract (web summary)

STIM proteins sense Ca^2+^ depletion in the ER and activate store-operated Ca^2+^ entry in response, a process associated with dendritic cell (DC) functions. Here, the authors show that optimal antigen cross-presentation in DCs requires the association of the chaperone molecule UNC93B1 with STIM1.

## Introduction

In professional antigen-presenting cells such as dendritic cells (DCs) or macrophages, exogenous antigens can be presented by MHC class I (MHCI) molecules, a process described as the cross-presentation pathway^1,2^. Cross-presentation has a fundamental function in the induction of CD8^+^ T cell immunity and controls immune response against pathogens and immune tolerance to self-antigens. Most work on cross-presentation has focused on DCs, in particular Xcr1^+^ DCs that express CD8 or CD103, as they are the predominant cells in vivo to cross-present antigens^3^. In DCs, exogenous antigens are partially proteolysed in endosomal/phagosomal compartments after uptake through endocytosis and phagocytosis. One particular feature of DCs is the slightly acidic pH of their cross-presentation compartments, which is generated by the late recruitment of the V-ATPase (the principal proton carrier in the lysosomes) that acidifies the lysosomes and the early recruitment of the NADPH oxidase 2 complex (NOX2), which slows down the acidification by consuming protons^4^. Thus, DCs express proteases with low activity when compared to macrophages^5,6^. This mild proteolytic environment prevents excess degradation of the antigen and thus facilitates transport of the antigen to the cytosol, another critical step for antigen cross-presentation in DCs^7,8^. In the cytosol, antigens are further degraded by the proteasome and the resulting peptides are either transported in the endoplasmic reticulum (ER) or back into the phagosomes via the transporter associated with antigen processing where they can be loaded on MHCI molecules. The last antigen-processing steps may follow different cellular routes involving the ER-associated amino peptidase in the ER and the insulin-responsive amino peptidase in the phagosome^9,10^.

Toll-like receptors (TLRs) bind conserved molecules from microorganisms, and in DCs are crucial in linking innate to adaptive immunity. Endosomal TLRs sense viral and bacterial nucleic acids such as double/single-stranded RNA or double-stranded DNA. Specific interaction between TLRs and their ligands results in induction of DC maturation, which boosts MHCI cross-presentation by a variety of mechanisms. TLR activation leads to (1) enhancement of antigen uptake by endocytosis and macropinocytosis, (2) MHCI recruitment to the phagosomes, (3) antigen translocation to the cytosol, and (4) reduction in the recruitment of active proteases to phagosomes^5,6,11,12^. Recently, Alloatti et al.^13^ have shown that lipopolysaccharide, a TLR4 ligand, increases antigen cross-presentation in DCs through delayed phagosomal–lysosomal fusion. The ER membrane protein uncoordinated 93 homolog B1 (UNC93B1) has an important function in regulating intracellular TLR signaling. The nucleic acid-sensing TLRs require UNC93B1 for trafficking from the ER to the endosomes where they are cleaved and activated^14–18^. Indeed, a single nucleotide mutation in *Unc93b1* gene (*Unc93b1* ^*3d*/*3d*^) abrogates the interaction with TLR3, 7, and 9, but also cross-presentation of antigens by MHCI molecules, even in the absence of TLR help^19^. However, the underlying molecular mechanisms leading to impaired cross-presentation are unclear. In addition, mice with the 3d mutation are not able to respond to mouse cytomegalovirus or *Listeria monocytogenes* because of impaired intracellular TLR activation^19^. Also, in humans two patients with autosomal recessive UNC93B1 deficiency have been described to be more susceptible to TLR3-dependent human herpes simplex virus infection, which results in impaired anti-interferon antiviral responses^20^. Altogether, these results underline the important role for UNC93B1 in viral infection due to impaired TLR sensing and exogenous antigen presentation.

The stromal interacting protein 1 (STIM1) is an ER resident protein that detects variation of calcium in the ER. Upon phagocytosis, Ca^2+^ depletion from the ER is sensed by STIM1 through its EF hand domain in the lumen of the ER, which results in STIM1 clustering and translocation to sections of the ER juxtaposed to the plasma membrane (PM), leading to ER-PM contact sites^21^. STIM1 will then recruit and activate ORAI1, one of the main store-operated Ca^2+^ entry (SOCE) channels resulting in localized Ca^2+^ influx. SOCE has been reported to be the major source of Ca^2+^ in DCs^22^. Some studies have described STIM1 to be critical for DC maturation, but STIM1 has also been reported to have no role in DC maturation and MHCII antigen presentation^23,24^. However, antigen cross-presentation was not assessed in these reports. The importance of STIM1 in lymphocyte function (especially in T cells) has been widely described and patients with loss of expression or mutations in STIM1 develop chronic infections such as herpes like viruses and severe combined immunodeficiency^25,26^.

Here, we show that UNC93B1 associates with STIM1 ER-luminal domain and helps its oligomerization state. In UNC93B1-mutated (3d/3d) DCs, endosomal/phagosomal pH is increased, phago-lysosome fusion, proteolytic activity and antigen export to the cytosol are decreased and the ability of STIM1 to interact with UNC93B1 and to activate membrane Ca^2+^ channels is diminished, causing a decrease in antigen degradation and impaired antigen cross-presentation. Furthermore, transfection of a cDNA coding for an active form of STIM1, which no longer associates with UNC93B1, restores antigen degradation and cross-presentation in 3d/3d DCs. Altogether, these results explain how the UNC93B1 protein, either alone or via its association with STIM1, controls cross-presentation of antigens in DCs, and demonstrate a critical role for STIM1 in this process.

## Results

### 3d mutation inhibits antigen cross-presentation

**Fig. 1:**
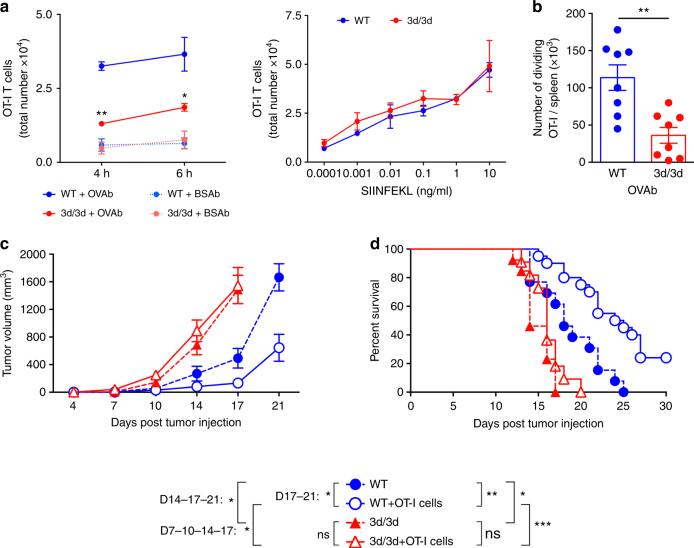
The UNC93B1 mutation 3d impairs antigen cross-presentation and tumor response. **a** CD11c^+^CD8^+^ cDCs purified from WT and 3d/3d mice were incubated with different concentrations of OVA beads (OVAb) or OVA peptide (SIINFEKL) for 4 or 6 h before co-cultured with CD8^+^ OT-I T cells for 72 h. T cell activation was monitored by evaluation of the total number of T cells. Graphs show mean ± S.E.M. (*n* = 3). Statistical significance was determined by unpaired *t*-test. *P < 0.05, **P < 0.01. **b** WT and 3d/3d mice were intravenously injected with CFSE-labeled OT-I T cells and 16 h later immunized with OVAb. T cell proliferation was monitored by CFSE dilution 72 h later. Graph shows the number of dividing OT-I T cells per spleen of immunized mice (*n* = 8 mice for WT or 3d/3d; **P < 0.01 via unpaired *t*-test; mean ± S.E.M.). **c**, **d** UNC93B1 sufficient (WT, circle) and deficient (3d/3d, triangles) mice were injected with B16-OVA cells (2 × 10^5^, s.c.) on day 0. When indicated, mice were adoptively transferred with OT-I T cells (2.5 × 10^6^, i.v. open symbols, plain lines) on day 1. Tumor size was measured every 2–4 days and survival was monitored daily. Data are illustrated as: **c** tumor volume (mean ± S.E.M.), **d** percentage of mice survival, and were pooled from three independent experiments (11–15 mice/group). Statistical analyses were done by Log-Rank (right, survival curves) and multiple *t*-tests (left, tumor volume); *P < 0.05; **P < 0.001, ***P < 0.001, ns for not significant

We first investigated, as previously described^19^, MHCI antigen cross-presentation in DCs from wild-type (WT) or UNC93B1-mutated (3d/3d) mice using ovalbumin (OVA) antigen. Purified spleen CD8^+^ CD11c^+^ DCs and bone marrow-derived DCs (BM-DCs) from WT or 3d/3d mice were incubated for 2, 4, or 6 h with different concentrations of OVA-coated beads (OVAb). Cells were then washed and co-cultured with CFSE-labeled OT-I T cells or B3Z hybridoma (both CD8^+^ T cells recognizing H-2K^b^-OVA complexes) and T cell activation was measured either by CFSE dilution or β-galactosidase activity. As expected, cross-presentation was dramatically reduced in 3d/3d DCs in comparison to WT cells (Fig. 1a and Supplementary Fig. 1a). The SIINFEKL peptide, which is recognized by OT-I and B3Z T cells and does not require any processing or proteasome degradation was presented equally by WT and 3d/3d DCs (Fig. 1a and Supplementary Fig. 1a). In addition, phagocytosis and MHCI expression were similar in WT and 3d/3d cells (Supplementary Fig. 1b, c). To assess cross-presentation in vivo, we injected WT and 3d/3d mice intravenously with CFSE-labeled OT-I T cells and 16 h later immunized them with OVAb. T cell proliferation was monitored 72 h after injection. We observed a decrease in the number of dividing OT-I T cells in the spleen of 3d/3d mice in comparison to WT mice (Fig. 1b). Cross-presentation is crucial for induction of adaptive response against tumors. To analyze UNC93B1-deficient and sufficient mice in the context of tumor progression, WT or 3d/3d mice received 2 × 10^5^ B16 OVA-expressing melanoma cells (B16-OVA) subcutaneously, 24 h before adoptively transferring or not 2.5 × 10^6^ OT-I T cells. In WT mice, OT-I cells induced a significant delay in tumor growth. In contrast, tumor progression in 3d/3d mice was unaffected by OT-I transfer and tumors reached a maximal size of 1500 mm^3^ on average at day 17 (Fig. 1c). In terms of survival, tumor-bearing WT mice having received OT-I cells displayed significantly prolonged survival than 3d/3d mice (Fig. 1d). Interestingly, we observed that tumors grew faster in 3d/3d mice than in WT mice in the absence of OT-I cells. In agreement with this, WT mice survived longer than 3d/3d mice (Fig. 1d), illustrating that inefficient anti-tumor mechanisms are present in 3d/3d mice^27^. We thus conclude that 3d/3d DCs in vitro and in vivo cross-present antigens significantly less efficiently than WT DCs.

### Impaired acidification and proteolysis in 3d/3d phagosomes

**Fig. 2:**
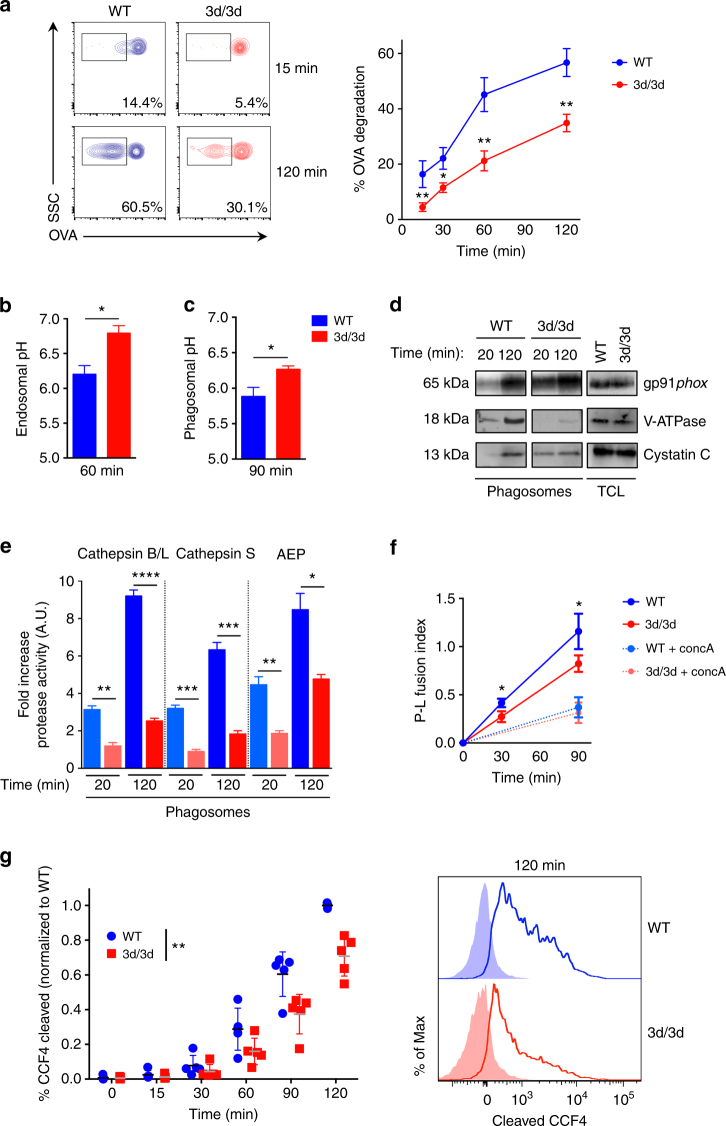
Phagosomal acidification and proteolysis are compromised in 3d/3d DCs. **a** BM-DCs were incubated with OVAb for 15 min (pulse), and cargo fate (OVA Alexa-488 staining) was monitored by flow cytometry on phagosomes for the indicated chase points. The data displayed on the left panel are representative of one experiment and on the right panel is the quantification of percentage of OVA degradation (mean ± S.E.M., *n* = 4; *P < 0.05; **P < 0.01 using unpaired *t*-test). **b** For endosomal pH measurement, WT and 3d/3d DCs were pulsed for 10 min with FITC and Alexa-647-coupled dextrans (40 kDa) and chased for 50 min. Graph shows mean ± S.E.M. (*n* = 3) *P < 0.05 by unpaired *t*-test. **c** Phagosomal pH (90 min) was measured using ratiometric imaging by exposing cells to FITC-coupled OVA-coated zymosan (mean ± S.E.M., *n* = 3, *P < 0.05 via unpaired *t*-test). **d** Phagosomes from WT or 3d/3d DC were purified after 20 min or 2 h of particle internalization. Protein expression of gp91*phox*, V-ATPase and Cystatin C was detected either in total lysate (TCL, 50 μg) or in phagosomes (5 μg). Data are representative of three experiments. **e** Proteases’ activity in early (20 min) or late (120 min) phagosomes from WT (blue histogram) and 3d/3d (red histogram) DCs were measured with specific fluorescent substrates. Graphs show mean ± S.E.M., *n* = 3, *P < 0.05, **P < 0.01, ***P < 0.001, ****P < 0.0001 using unpaired *t*-test. **f** Phago-lysosome fusion (P-L) was measured by exposing cells loaded with lysosomal FRET acceptor Alexa-594-HA to donor Alexa-488 OVAb (*n* = 4). Cells were treated or not with ConcA as a negative control(*n* = 2) (*P < 0.05, unpaired *t*-test mean ± S.D.). **g** Percentages of β-lactamase transport to the cytosol were measured by loading WT and 3d/3d DCs with CCF4 dye followed by incubation with β-lactamase for the indicated time points (left panel, each dot corresponds to one experiment, *n* = 5). Representative histogram (right panel) of CCF4 cleaved product at 120 min in WT (blue solid line) and 3d/3d (red solid line). DCs incubated with CCF4 alone are shown in dashed lines **P < 0.01 by two-way ANOVA comparing all time points

UNC93B1 is recruited in LAMP1^+^ compartment after 2 h of phagocytosis (Supplementary Fig. 2a), indicating that it might play a role in phagosomal compartment. To investigate how 3d/3d DCs have a decreased capacity to cross-present antigens, we first monitored the degradation of OVA using a flow cytometry assay. OVAb were internalized by phagocytosis for different time points and OVA degradation in phagosomes was assessed with anti-OVA staining. In WT DCs, OVA degradation increased upon time as expected (Fig. 2a). In contrast, 3d/3d phagosomes displayed reduced antigen degradation. To determine whether the decrease in OVA degradation in 3d/3d phagosomes reflected a change in endosomal/phagosomal acidification, we measured endosomal pH at 60 min after DCs incubation with 40 kDa dextrans coupled to pH-sensitive (FITC) or to pH-insensitive dyes (Alexa-647)^16^. Phagosomal pH was quantified via ratiometric imaging by exposing cells to FITC-coupled OVA-coated zymosan, as previously described^28,29^. Both endosomes and phagosomes from 3d/3d BM-DCs had increased pH compared to WT cells (Fig. 2b, c). To address whether higher pH in 3d/3d BM-DCss was dependent on NOX2, we monitored its expression and activity in phagosomes^30^. Early (20 min) and late phagosomes (120 min) from WT and 3d/3d mice were purified using magnetic beads and expression of gp91*phox*, one of the NOX2 subunits, was detected by immunoblot^16^. As shown in Fig. 2d, early phagosomes from 3d/3d DC express more gp91*phox* compared to WT phagosomes. In contrast, comparable gp91*phox* levels were detected in late phagosomes and in total cell lysates from both WT and 3d/3d DCs. To measure phagosomal ROS (NOX2 activity), BM-DCs were challenged with OVA-opsonized zymosan coupled to OxyBurst and Alexa-568 and the ratio of OxyBurst to Alexa-568 fluorescence was detected over time by immunofluorescence^29^. After 30 min of phagocytosis, ROS activity was found in WT and 3d/3d phagosomes and increased over time. However, we noticed a slight but significant increase in phagosomal ROS from 3d/3d DCs in comparison to WT (Supplementary Fig. 2b). As expected, ROS production was inhibited by diphenylpropidium iodide (DPI), a NOX2 inhibitor, demonstrating that phagosomal ROS production is NOX2 dependent (Supplementary Fig. 2b). We confirmed that total ROS production remained the same between WT and 3d/3d DCs and was also inhibited by DPI (Supplementary Fig. 2c). Additionally, V-ATPase recruitment was both delayed and decreased in 3d/3d phagosomes confirming the reduced acidification in these cells (Fig. 2d). As OVA degradation and pH are linked to phagosomal proteolytic activity, we isolated early and late phagosomes and assessed the activity of lysosomal proteases with specific synthetic fluorescent substrates^16^. As expected, cathepsins B/L, S and asparagine endopeptidase (AEP) activities were increased in late phagosomes vs. early phagosomes, reflecting the progressive acidification of this organelle. However, a strong decrease in their proteolytic activities was detected in 3d/3d phagosomes in comparison to WT phagosomes (Fig. 2e). This result correlated with increased cystatin C expression, a cysteine protease inhibitor, in early phagosomes from 3d/3d DCs (Fig. 2d). The late recruitment of the V-ATPase in 3d/3d phagosomes might suggest a defect in phago-lysosome fusion. To address this, BM-DCs were pulsed with the FRET acceptor Alexa-594-HA for 3 h and chased for 16 h to accumulate the dye in lysosomes. BM-DCc were then challenged with OVAb linked to the FRET donor Alexa-488 for 30 and 90 min and imaged by fluorescence microscopy. As shown in Fig. 2f, phago-lysosome fusion was significantly reduced in 3d/3d BM-DCs. Also, the increased recruitment of gp91*phox* in early 3d/3d phagosomes was concomitant with higher Rab27a levels in comparison to early WT phagosomes (Supplementary Fig. 2d), suggesting the differential recruitment of distinct “inhibitory lysosome-related organelles” containing NOX2 subunits in 3d/3d phagosomes, previously shown to regulate phagosomal pH and ROS activity^31^.

Antigen export to the cytosol has been previously shown to be an important event for antigen cross-presentation^8^. To investigate antigen export to the cytosol we used an established assay based on the enzymatic activity of β-lactamase^32^. DCs were loaded with the probe CCF4 for 1 h and β-lactamase was added to the cells for different times. The β-lactamase is internalized through endocytosis and when it is exported in the cytosol it cleaves the CCF4 probe, which fluoresces at 450 nm. In WT cells, β-lactamase activity increased over time reflecting the increase in antigen export to the cytosol. However, as displayed in Fig. 2g, β-lactamase activity was significantly delayed in 3d/3d DCs. We concluded that UNC93B1 is important for phagosomal maturation and plays an active role in regulating endo/lysosomal pH, leading to reduced proteolytic activity and antigen export to the cytosol.

### Expression of STIM1 is downregulated in 3d/3d spleen cells

**Fig. 3:**
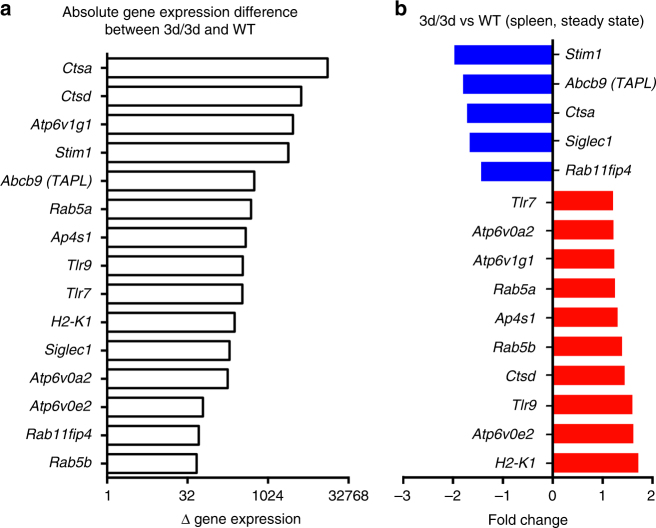
Expression of *Stim1* mRNA is downregulated in 3d/3d spleen cells. Histograms show **a** the absolute gene expression difference between 3d/3d and WT mice for proteins involved in endolysosomal maturation and antigen presentation and **b** the relative fold changes in gene expression in 3d/3d vs. WT splenocytes (genes upregulated are shown in red and downregulated in blue)

To investigate if UNC93B1 itself or/and via the association with other protein(s) contributes to MHCI antigen cross-presentation, we analyzed the expression of genes involved in antigen cross-presentation in spleen cells from WT and 3d mice using the publicly available microarray database (GSE41496). We found that among highly expressed antigen-presentation-related genes (coding for cathepsin D, TAPL, V-ATPase subunits), the calcium sensor *Stim1* gene was one of the most highly expressed in WT spleen (Fig. 3a) but also the most downregulated in 3d/3d spleen compared to WT (Fig. 3b). To validate these data, we measured STIM1 protein expression in the main spleen cell subsets, namely, B cells, T cells, and DCs^33^. As shown in Supplementary Fig. 3a, STIM1 was not expressed in B cells from WT or 3d/3d mice and less STIM1 was detected in 3d/3d spleen T cells. However, similar expression of STIM1 was found in cDCs and BM-DCs from WT and 3d/3d mice (Supplementary Fig. 3b). These results suggest that impaired antigen cross-presentation in 3d/3d DCs is not due to decreased STIM1 expression.

### STIM1 function and Ca^2+^ influx are impaired in 3d/3d DCs

**Fig. 4:**
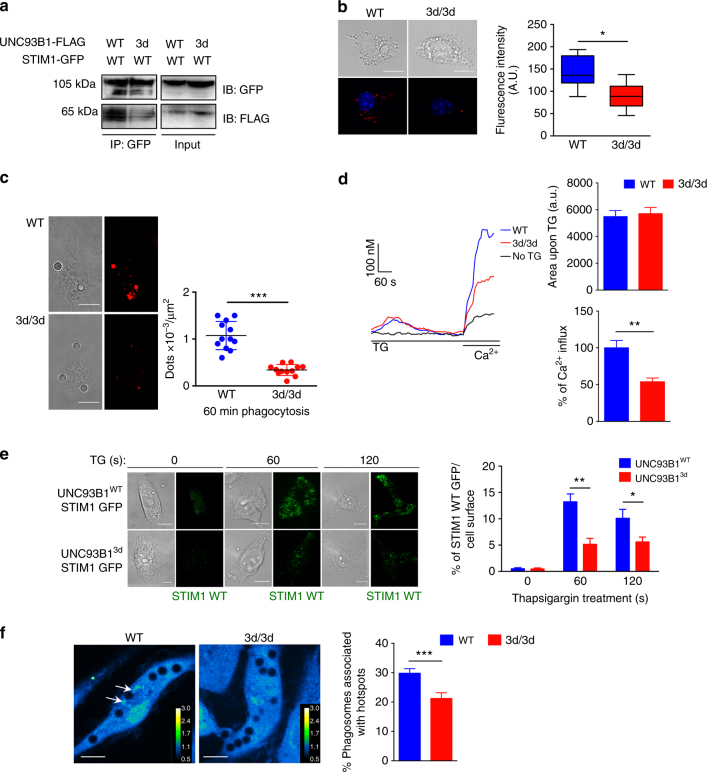
Reduced Unc93b1^3*d*^-STIM1 association compromises STIM1 function. **a** Fibroblasts expressing STIM1-WT-GFP-tagged and UNC93B1^WT^ or UNC93B1^3d^-FLAG-tagged, plasmids were lysed and STIM1 was immunoprecipitated with GFP beads and immunoblotted with anti-GFP and anti-FLAG antibodies. **b** In WT and 3d/3d DCs transfected with STIM1-WT-GFP-plasmid, STIM1–UNC93B1 interaction was detected using Duolink proximity ligation assay with anti-GFP and anti-UNC93B1-specific antibodies. Cells are seen in brightfield (top panel). PLA signals are shown in red and nuclei in blue (bottom panel) and quantified with Image J (*n* = 11 cells; *P < 0.05). Bars = 10 μm. **c** Representative figures and quantification of PLA signals (dots/μm^2^) after 60 min of phagocytosis of 3 μm beads by WT and 3d/3d DCs transfected with STIM1-WT-GFP cDNA (*n* = 12 cells; ***P < 0.001). Bars = 10 μm. **d** Averaged Ca^2+^ signals in WT and 3d/3d DCs loaded with fluo4-AM as described in Methods section. Cells were stimulated with 1 μM TG (thapsigargin) in Ca^2+^-free medium before addition of 2 mM Ca^2+^ for the time periods indicated by the horizontal bars. One representative experiment is presented. Histograms summarize five separate experiments (triplicates) showing the area of the fluorescence signal under TG-induced response in the absence of external Ca^2+^ and percentage of influx following addition of 2 mM Ca^2+^ (mean ± S.E.M.; **P < 0.01). **e** Fibroblasts were co-transduced with STIM1-WT-GFP- and UNC93B1^WT^ or UNC93B1^3d^-Cherry-tagged plasmids followed by treatment with TG in Ca^2+^-free Ringer’s solution for the time points indicated or left untreated. Representative TIRF images of STIM1-WT-GFP (left panel) and quantification of percentage of fluorescence intensity per cell area using Image J (*n* = 15 cells; *P < 0.05; **P < 0.01). Bars = 10 μm. **f** Localized Ca^2+^ traces near phagosomes (green dots, white arrow) were measured in WT and 3d/3d DC loaded with 4 μM Fluo-8 beads for 30 min uptake and 2.5 μM BAPTA. The color-coded ratio images represent Fluo-8 fluorescence divided by the average cytosolic Fluo-8 fluorescence (left panels) and quantification shows percentage of periphagosomal Ca^2+^ hotspots (*n* = 4 experiments/462–660 phagosomes for WT and 136–200 phagosomes for 3d/3d DCs) (right panel; mean ± S.E.M.; ***P < 0.001). Bars = 10 μm. For **a**, **b**, **c**, **e** one experiment out of three is shown. Statistics are performed via unpaired *t*-test

The majority of intracellular Ca^2+^ is sequestered within the ER, and Ca^2+^ signaling near phagosomes either through release of ER Ca^2+^ or through SOCE promotes phagosome maturation. STIM1 is an ER calcium sensor that was shown to be recruited near phagosomes to generate Ca^2+^ hotspots important for phagocytosis^29^. Even though the expression of STIM1 was not reduced in 3d/3d DCs, we reasoned that STIM1 function might be altered. First, we assessed UNC93B1-Stim1 possible interaction by co-transfecting Stim1-GFP and Unc93b1^WT^-FLAG or Unc93b1^3*d*^-FLAG cDNAs in fibroblasts followed by immunoprecipitation of overexpressed UNC93B1-tagged and STIM1-tagged proteins. We found that STIM1 interacted with WT UNC93B1 but 3d mutation reduced this interaction (Fig. 4a). Likewise, 3d-UNC93B1 was associating less with STIM1 compared to WT-UNC93B1 (Supplementary Fig. 4a). The rates of transfection of Stim1-GFP and Unc93b1^WT^-FLAG or Unc93b1^3*d*^-FLAG cDNAs were identical (Supplementary Fig. 4b). We confirmed endogenous STIM1/UNC93B1 interaction in BM-DCs (Supplementary Fig. 4c). We then performed proximity-ligation assay, which monitors protein–protein interaction in situ^34–36^. Confocal microscopy indicated that indeed less 3d protein was detected in proximity to STIM1 at the steady state (Fig. 4b) and upon phagocytosis (Fig. 4c) when compared to WT UNC93B1. Second, to analyze the function of STIM1 in WT and 3d/3d DCs, we monitored Ca^2+^ flux. ER Ca^2+^ was depleted using thapsigargin (TG) (the sarcoplasmic ER Ca^2+^ ATPase inhibitor) and extracellular Ca^2+^ was added to fluo4-loaded cells. In WT cells, we observed a large Ca^2+^ influx (greater than in the absence of TG, probably due to activation of cells in medium deprived of Ca^2+^), which was reduced by 50% in 3d/3d DCs (Fig. 4d). However, the area under the curve, representing ER Ca^2+^ release upon TG stimulation was similar between WT and 3d/3d cells strongly suggesting that ER-Ca^2+^ load was unaffected (Fig. 4d). Similarly, live imaging in Fura 2-AM-loaded DCs revealed absence of Ca^2+^ influx in situ in 3d/3d DCs treated as in Fig. 4d (Supplementary Fig. 4d) in comparison to WT DCs. Upon ER Ca^2+^ depletion, STIM1 moves to the PM where it is juxtaposed to ORAI1 that, in turn, opens up and allows the extracellular Ca^2+^ to enter the cells. We thus hypothesized that STIM1 transfer into ER-PM junctions might be also altered by the 3d mutation. To analyze STIM1 apposition to the PM, we performed TIRF microscopy in fibroblasts transfected with Stim1-GFP and mutated or WT Unc93b1-cherry cDNAs and treated with TG for 60 or 120 s. As shown in Fig. 4e, significantly less STIM1-GFP reached the PM in fibroblasts expressing mutated UNC93B1 than WT UNC93B1. In macrophages and neutrophils, localized Ca^2+^ hotspots were previously described upon phagocytosis^29^ and in neutrophils it correlated with STIM1-dependent apposition of ER membranes to phagosomes. We confirmed that periphagosomal Ca^2+^ hotspots could be detected in WT DCs. In contrast, in 3d/3d DCs, a significant decrease in Ca^2+^ hotspots was observed upon phagocytosis (Fig. 4f). Altogether, these results imply that UNC93B1 interacts with STIM1 and that in 3d/3d DC STIM1 function is compromised.

### UNC93B1 is important for STIM1 oligomerization

**Fig. 5:**
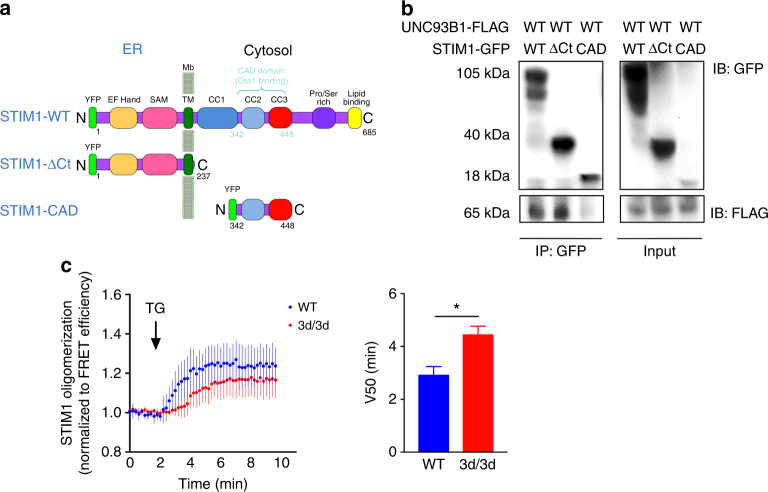
UNC93B1 association with STIM1 ER-luminal is required for STIM1 oligomerization. **a** Schematic representation of STIM1 full-length protein (WT) and STIM1 mutants (STIM1-ΔCt and STIM1-CAD) used in this study. **b** Fibroblasts expressing GFP-tagged STIM1- WT, −ΔCT and −CAD and UNC93B1^WT^-FLAG-tagged plasmids were immunoprecipitated with GFP beads and immunoblotted with anti-GFP and anti-FLAG antibodies. One experiment representative out of two is shown. **c** STIM1 oligomerization was followed in HeLa cells in the presence of either Cherry-tagged WT or 3d-mutated UNC93B1 by measuring the increase in the FRET efficiency of YFP-tagged and CFP-tagged STIM1 upon stimulation with 1 μM TG over time. The V50 parameter is reported as the time-to-half–maximum FRET efficiency. Graph shows mean ± S.E.M. (*n* = 3 experiments); *P < 0.05 by paired *t*-test

To identify the site of the STIM1/UNC93B1 interaction, we co-transfected Stim1-GFP mutants (Fig. 5a) and Unc93b1^WT^-FLAG cDNAs in fibroblasts and STIM1/UNC93B1 complexes were then immunoprecipitated. As shown in Fig. 5b, both STIM1-WT and STIM1-ΔCt (a mutant of STIM1 expressing the N-terminal domain localized in the ER) interacted with WT UNC93B1. No association was observed between UNC93B1 and STIM1-CAD, a mutant of STIM1 expressing solely the cytosolic CAD domain, which binds and activates ORAI1 (Fig. 5b). The luminal or N-terminal domain of STIM1 is composed of two EF-hand motifs, one of which binds Ca^2+^ and, a SAM domain^37^ (Fig. 5a). At the steady state, STIM1 binds Ca^2+^ and there is a stable EF-hand-SAM configuration. When Ca^2+^ is depleted, the EF-SAM domain unfolds and STIM1 rapidly oligomerizes (puncta staining). To monitor whether STIM1 oligomerization was impaired in 3d/3d cells, we performed FRET experiments in HeLa cells transiently expressing CFP-tagged and YFP-tagged STIM1 together with UNC93B1-cherry (WT or 3d). Our results revealed that in the presence of 3d protein, STIM1 oligomerized less and more slowly (Fig. 5c). Altogether, these results indicate that UNC93B1 interacts with STIM1 luminal domain and that STIM1 oligomerization is decreased in cells expressing the 3d mutation.

### Silencing STIM1 in DCs inhibits antigen cross-presentation

**Fig. 6:**
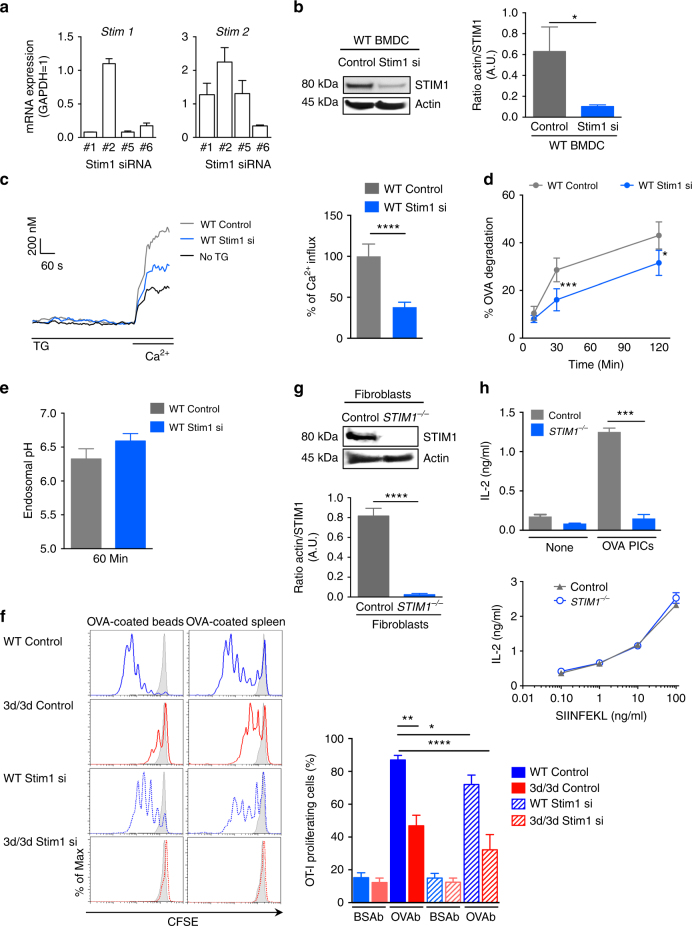
Impaired antigen degradation and cross-presentation in cells with STIM1 ablation. **a** Quantitative PCR analysis of *Stim1* and *Stim2* gene expression in DCs nucleofected with Stim1-specific siRNA (mean ± S.E.M.; *n* = 3 experiments). **b** Immunoblot of STIM1 and actin expression in WT (control siRNA) and Stim1-silenced (siRNA #5) DCs and quantification of actin/STIM1 ratio of three independent experiments (**P < 0.01 via unpaired *t*-test). **c** Averaged Ca^2+^ signals in control and STIM1-silenced DCs loaded with fluo-4AM and stimulated with TG in Ca^2+^-free solution before adding 2 mM Ca^2+^. One representative experiment is shown (left panel) (*n* = 3 × 10^5^ cells) and graph shows Ca^2+^ influx (right) from three independent experiments normalized to siRNA control cells (****P < 0.0001 via paired *t*-test). **d** Proteolysis was measured by quantifying the degradation of OVA in control and STIM1-silenced phagosomes for the indicated time points (*n* = 3 experiments; mean ± S.E.M.; *P < 0.05, ***P < 0.001 via paired *t*-test). **e** Endosomal pH in control and STIM1-silenced DCs pulsed for 10 min with FITC-coupled and Alexa-647-coupled dextrans and chased for 50 min (*n* = 3; mean ± S.E.M.). **f** DCs knocked down for STIM1 or not from WT and 3d/3d mice were challenged with BSAb, OVAb, or OVA-transfected splenocytes as sources of exogenous antigen before co-culture with CFSE-labeled OT-I T cells. T cell proliferation was monitored by flow cytometry 3 days later. Representative histogram plots are shown (left). Quantification (right) of OT-I cell division is shown as mean percentage of proliferating cells (*n* = 3; mean ± S.E.M.; *P < 0.05; **P < 0.01; ****P < 0.0001 using unpaired *t*-test). **g** Immunoblot analysis of STIM1 and actin protein expression in control human fibroblasts and fibroblasts from a patient with STIM1 deficiency and quantification of actin/STIM1 ratio. ****P < 0.01; *n* = 3 (unpaired *t*-test). **h** Control and STIM1-deficient FcγRIIA-EGFP-K^b^-transduced human fibroblasts were stimulated with precipitated OVA immunocomplexes (OVA pICs) or BSA pICs (none) as control (upper panel) or SIINFEKL peptide (lower panel) before co-culture with B3Z hybridoma. IL-2 secretion was measured by ELISA (*n* = 3; mean ± S.E.M.; ***P < 0.001 via unpaired *t*-test)

To investigate the contribution of STIM1 in cross-presentation, we knocked down the expression of STIM1 in BM-DCs using four different siRNA, with siRNA #1 and #5 being the most potent at reducing *Stim1* mRNA levels without interfering with *Stim2* mRNA (Fig. 6a). Moreover, using Stim1 siRNA #5, STIM1 protein expression was reduced by 80% (Fig. 6b). To monitor whether the function of STIM1 in cells silenced for STIM1 was abrogated, we assessed calcium flux in BM-DCs transfected with Stim1 siRNA #5. In WT cells, we observed a Ca^2+^ influx that was reduced by 50% in DCs silenced for STIM1 (Fig. 6c), similarly to what we found in 3d/3d DCs (Fig. 4d). We then assessed if loss of endogenous STIM1 could modulate antigen processing and cross-presentation by monitoring endosomal pH, OVA degradation, and OT-I proliferation, as described above. In BM-DCs silenced for STIM1, OVA was degraded less, which correlated with a slight increase, yet not significant, in endosomal pH (Fig. 6d, e). To assess antigen cross-presentation, DCs from WT and 3d/3d mice transfected with control (non-targeting) or *Stim1* siRNA were incubated with OVAb or OVA-coated splenocytes, co-cultured with CFSE-labeled OT-I T cells and T cell activation was measured. As expected, cross-presentation was reduced in 3d/3d DCs in comparison to WT cells, both expressing control siRNA. Knockdown of STIM1 resulted in a significant decrease in antigen cross-presentation, whereas SIINFEKL presentation was not affected (Fig. 6f and Supplementary Fig. 5a). Furthermore, silencing STIM1 in 3d/3d cells abolished cross-presentation (Fig. 6f and Supplementary Fig. 5a). To test whether or not human cells lacking STIM1 had a defect in antigen cross-presentation, we obtained fibroblasts from a patient (patient V-1) with recessive nonsense mutation in *Stim1* gene, resulting in premature termination of codon^25^. Expression of STIM1 was not detected in patient V-1 fibroblasts compared to control fibroblasts, as anticipated (Fig. 6g). To establish a system competent for cross-presentation in non-professional antigen-presenting cells^38^, we co-transfected fibroblasts with GFP-tagged human FcγRIIA together with the mouse MHCI (H-2K^b^) molecule. Expression of H-2K^b^ and FcγRIIA-GFP was similar in control and STIM1-deficient fibroblasts (Supplementary Fig. 5b). Cells were incubated with precipitated OVA immune complexes (OVA-pICs) uptaken by FcγRIIA-dependent phagocytosis, washed and co-cultured with B3Z T cell hybridoma. As shown in Fig. 6h, fibroblasts lacking STIM1 were unable to cross-present OVA-pICS, while SIINFEKL peptide was presented equally by both control and STIM1-deficient fibroblasts (Fig. 6h). These results strongly suggest that STIM1 is required for OVA degradation and cross-presentation in murine and human cells.

### Active STIM1 restores cross-presentation in 3d/3d cells

**Fig. 7:**
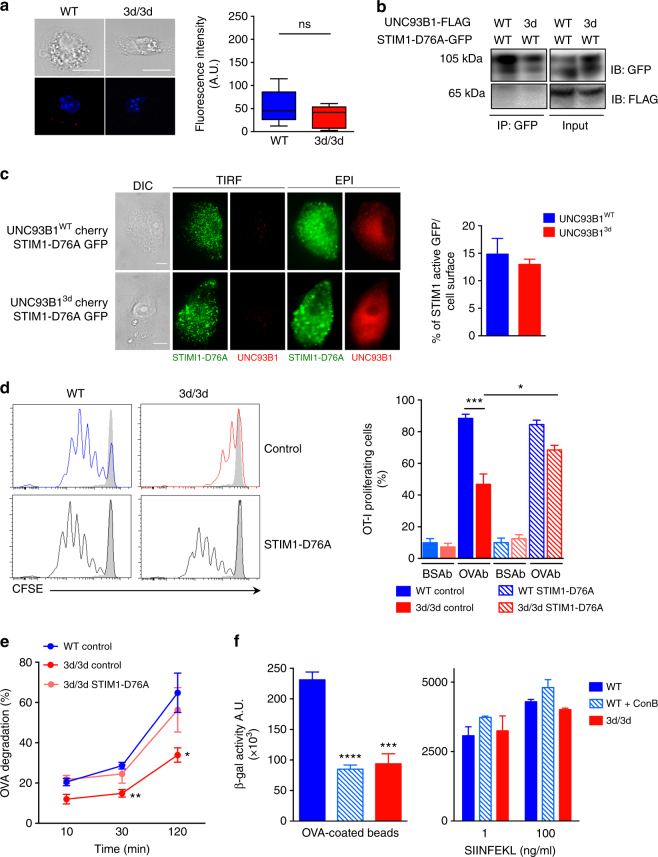
Active STIM1 restores antigen proteolysis and cross-presentation in 3d/3d DCs. **a** Detection of STIM1 active (STIM1-D76A) and UNC93B1 interaction using Duolink proximity ligation assay (PLA) with anti-GFP (STIM1-D76A) and anti-UNC93B1-specific antibodies in WT or 3d/3d DCs. Quantification of mean fluorescence using ImageJ software (*n* = 12 cells; mean ± S.E.M.; ns for non-significant). One experiment out of three is shown. Bars = 10 μm. **b** Fibroblasts expressing GFP-tagged STIM1-D76A and WT or 3d-mutated UNC93B1-FLAG-tagged plasmids were immunoprecipitated with GFP beads (STIM1-D76A) and immunoblotted with anti-GFP and anti-FLAG antibodies. One experiment representative out of two is shown. **c** Fibroblasts were transiently co-transduced with STIM1-D76A-GFP and WT or 3d-mutated UNC93B1-Cherry-tagged plasmids. Representative TIRF and EPI images of STIM1 active and UNC93B1 (left panel) and quantification of percentage of GFP fluorescence intensity per cell area using ImageJ software (*n* = 15 cells). One experiment out of two is shown. Bars = 10 μm. **d** DCs from WT and 3d/3d mice, transfected with STIM1-D76A-GFP or control GFP plasmids, were challenged with OVAb for 6 h before co-culture with CFSE-labeled OT-I T cells. T cell proliferation was monitored by flow cytometry 3 days later (*n* = 3; mean ± S.E.M.; *P < 0.05, ***P < 0.001 via unpaired *t*-test). **e** Phagosomal OVA degradation was measured at different time points in WT and 3d/3d DCs transfected with control GFP or with STIM1-D76A-GFP plasmids (*n* = 3 experiments; mean ± S.E.M.; *P < 0.05, **P < 0.01 via unpaired *t*-test). **f** WT and 3d/3d DCs were treated with ConB (20 nM) 10 min prior addition of OVAb (left panel) or SIINFEKL peptide (right panel) for 6 h. Cells were then extensively washed and co-cultured with B3Z CD8^+^ T cell hybridoma. T cell activation was monitored by measuring β-galactosidase activity (*n* = 3 experiments; mean ± S.E.M.; ***P < 0.001, ****P < 0.0001 via unpaired *t*-test)

We then examined if reconstitution of STIM1 activity in 3d/3d DCs could restore antigen cross-presentation. To address this, we transfected WT and 3d/3d DCs with GFP-tagged, constitutively active form of STIM1 (STIM1-D76A) assuming that STIM1-D76A would bypass the help of UNC93B1 for its function. We observed that STIM1-D76A was in lesser proximity to UNC93B1 protein (Fig. 7a) when compared with STIM1-WT (Fig. 4b) and we confirmed that it translocated constitutively to the PM independently of UNC93B1 (Fig. 7c). In addition, interaction between STIM1-D76A and UNC93B1 was lost (Fig. 7b), indicating that active STIM1 no longer required UNC93B1 for its oligomerization and juxtaposition to the PM to activate ORAI1. Nevertheless, phagocytosis and DCs activation were similar in WT and 3d/3d cells expressing or not the active form of STIM1 (Supplementary Fig. 6a, b). We then assessed antigen cross-presentation in DCs expressing STIM1-D76A. DCs were incubated with OVAb or BSAb and co-cultured with CFSE-labeled OT-I T cells. As shown in Fig. 7d, cross-presentation was not significantly increased in WT DCs expressing STIM1-D76A in comparison to WT cells transfected with an empty vector. More importantly, cross-presentation was restored in 3d /3d DCs in the presence of constitutively active STIM1 (Fig. 7d). The rescue in cross-presentation observed in 3d/3d cells correlated with an increase in OVA degradation to nearly the same level as the one observed in WT cells (Fig. 7e). Also, the use of concanamycin B (ConB), a V-ATPase inhibitor blocking endosomal/phagosomal acidification^16^, inhibited antigen cross-presentation in WT DCs. Indeed, the defect in cross-presentation seen in WT cells treated with ConB was comparable to 3d/3d DCs (Fig. 7f). SIINFEKL peptide presentation was identical in WT, WT incubated with ConB and 3d/3d DCs indicating that ConB treatment does not interfere with antigen uptake or MHCI expression and trafficking. We conclude that interfering with antigen degradation either by restoring STIM1 activity in 3d/3d cells or by inhibiting endosomal acidification upon ConB addition in WT DCs has a major impact in antigen cross-presentation.

## Discussion

UNC93B1 is an ER resident protein described to be a regulatory subunit of a potassium channel in *Caenorhabditis elegans* ^39,40^. Yet, no function was assigned in mammals until mice with a single mutation in *Unc93b1* gene were generated using the random germline mutagen N-ethyl-N-nitrosourea^19^. This mutation disrupted TLR3, 7, and 9 signaling. Indeed, UNC93B1 was shown to physically interact with endosomal TLRs by co-immunoprecipitation studies in metabolically labeled cells and this association was lost in the 3d/3d cells^41^. Besides impaired TLR function, 3d/3d mice had also a decrease in MHC II antigen presentation and almost a total lack of antigen cross-presentation, which makes UNC93B1 protein an essential component of both innate and adaptive immunity. These results were unexpected because in 3d/3d mice the inhibition in exogenous antigen presentation was not linked to endosomal TLR activation.

In this study, in agreement with the group of B. Beutler^19^, we confirmed the defect in antigen cross-presentation in 3d/3d mice and cells. Furthermore, by using co-immunoprecipitation and PLA experiments, we identified a novel protein interacting with UNC93B1, the ER Ca^2+^ sensor STIM1 that regulates SOCE. SOCE influx is a fundamental mechanism of cell physiology not only by controlling the replenishing of intracellular Ca^2+^ stores, but also by regulating Ca^2+^-dependent enzymatic activity^42^, gene transcription^43^, and release of cytokines^44^. STIM1 forms homo-oligomers and hetero-oligomers and has a single transmembrane domain and a putative luminal EF-hand Ca^2+^-binding domain^45–47^. Upon ER calcium depletion, STIM1 promotes the formation of tight membrane-ER junctions known as cortical ER from where it interacts directly with Ca^2+^-permeable channels at the PM such as ORAI1^48,49^.

We found that a STIM1 mutant expressing only the ER luminal domain still binds UNC93B1 indicating that the interaction between these two proteins takes place in the ER. Additionally, we showed that a mutation in the Ca^2+^ EF-hand motif (STIM1-D76A), that renders STIM1 constitutively active, abrogates the interaction with UNC93B1, suggesting that the association between the two proteins requires STIM1 Ca^2+^-binding domain. The findings that constitutively active STIM1 can bypass the requirement for UNC93B1 binding for its translocation to the PM and that STIM1 oligomerized slower and less in 3d/3d cells indicate that UNC93B1 might be acting as a chaperone that aids in the early steps of STIM1 oligomerization. Future work delineating the exact mechanism through which UNC93B1 regulates STIM1 protein activation may yield new insights in the mechanism of this crucial signaling pathway.

Both UNC93B1-mutated and STIM1-silenced DCs displayed reduced global or local Ca^2+^ signals and decreased antigen proteolysis. Moreover, loss of STIM1 in murine DCs or in human fibroblasts resulted in decreased antigen cross-presentation. In addition, expression of an active form of STIM1 in 3d/3d DCs restored antigen degradation and cross-presentation. Altogether, these results reveal an important role for STIM1 in MHCI antigenic presentation pathway.

It has been shown that deletion of STIM1 using siRNA strongly diminishes the number of ER-PM junctions and that STIM1 over-expression leads to increased cortical ER formation upon TG ER-Ca^2+^ store depletion^48^. In a more recent study, Nunes et al.^29^ proposed that STIM1 activity is not strictly limited to the PM but occurred at the phagosomes. They suggested that STIM1 could also recruit the thin cortical ER to the phagosomes where it would also associate with ORAI1 and induce Ca^2+^ influx. The molecular mechanisms controlling antigen cross-presentation are still not well understood but many reports have suggested that phagosomes acquire the ER machinery needed for antigen cross-presentation, through ER-mediated phagocytosis, a process where ER membranes are recruited to the PM upon phagocytosis to form phagosomes^50–52^. Although direct fusion with the ER has been contested^53^, depletion of the ER-Golgi SNARE Sec-22b which blocks the delivery of ER proteins to phagosome led to an accelerated lysosomal recruitment^32^, thus inhibiting antigen cross-presentation and further indicating that ER-phagosome communication is required for MHCI cross-presentation. In agreement with this, slowing down phago-lysosomal fusion has been recently described to promote antigen cross-presentation^13^. Also, other vesicular fusion events could account for the transfer of ER components to phagosome^12,54^. Importantly, Nunes et al.(doi:10.1038/s41467-017-01601-6) showed that in DCs, STIM1 promotes the formation of contact sites between ER membranes and single phagosomes. Here, we demonstrate that UNC93B1 traffics to phagosomes and is in proximity to STIM1. Thus, it would be tempting to speculate that UNC93B1 is escorting STIM1 to phagosomes to exert its function. STIM1 could promote antigen cross-presentation by generating phagosomal Ca^2+^ hotspots, and promoting vesicle fusion events that might be required for the recruitment of ER proteins to phagosomes and for phago-endosomal or phago-lysosomal fusion. Indeed, we and Nunes et al. (doi:10.1038/s41467-017-01601-6) report a decrease in phago-lysosomal fusion in STIM1-deficient and 3d/3d DCs suggesting that Ca^2+^ plays a central role in this process.

Although this study identifies STIM1 being partly responsible for the decrease in antigen degradation and cross-presentation observed in 3d/3d cells and mice, our data indicate that in DCs UNC93B1, independently of STIM1, participates to phagosomal acidification, NOX2 recruitment and activity and antigen export to the cytosol, three important factors involved in antigen cross-presentation. Phagosomal ROS production by the Rac2-mediated recruitment of the NADPH oxidase, NOX2, is needed in DCs for efficient cross-presentation^30^. STIM1 controlled calcium influx in the vicinity of the PM and phagosomes and thus could interfere in the production of phagosomal ROS which was previously described to be affected in human and mouse STIM1-deficient cells^29,55^. However, it was not affected in STIM1-deficient DCs (doi:10.1038/s41467-017-01601-6). In contrast, we reported that NOX2 recruitment and ROS production were increased in 3d/3d phagosomes, which was consistent with higher Rab27a expression in 3d/3d phagosomes. Overall, our results suggest that the 3d mutation in DCs results in increased mobilization of a distinct subset of lysosome-related organelles containing membrane subunits of NOX2 that could account for limited acidification and degradation of internalized antigens^31^.

Phagosomal pH and proteolysis are important factors that regulate antigen cross-presentation. Several groups have shown that limited antigen degradation directly correlates with efficient cross-presentation^56,57^. In addition, recent studies have shown that the transcription factor TFEB regulates antigen processing by modifying lysosomal proteolysis activities and phagosomal acidification. In DCs, TFEB activity is low which correlates with high phagosomal pH and low proteolytic activity^58^. So, it is possible that UNC93B1 might regulate pH acidification through TFEB function. However, in our studies we observed a strong reduction in antigen cross-presentation in 3d/3d DCs, despite higher phagosomal pH and reduced proteolytic activity in phagosomes. A study from the group of RM Yates demonstrated that phagosomal NOX2 reduces antigen proteolysis through redox modulation of cysteine proteases in a pH-independent manner^59^, further reflecting the high level of complexity of the molecular mechanisms controlling cross-presentation. Indeed, 3d/3d DCs display dramatically reduced proteases activity together with increased NOX2 activity that both could account for reduced antigen processing. Altogether, we suggest that the fine-tuning between phagosomal acidification and antigen degradation and the orchestrated regulation of phagosomal maturation must be in place in DCs to allow for efficient antigen cross-presentation. Importantly, our results indicate that restoring one important parameter, such as antigen degradation, in 3d/3d DCs, by expressing an active STIM1 protein, is sufficient for effective cross-priming of naïve CD8^+^ T cells. However, we cannot exclude that the expression of STIM1 active form in 3d/3d DCs is not modifying other parameters, calcium-dependent or not, important for cross-presentation.

**Fig. 8:**
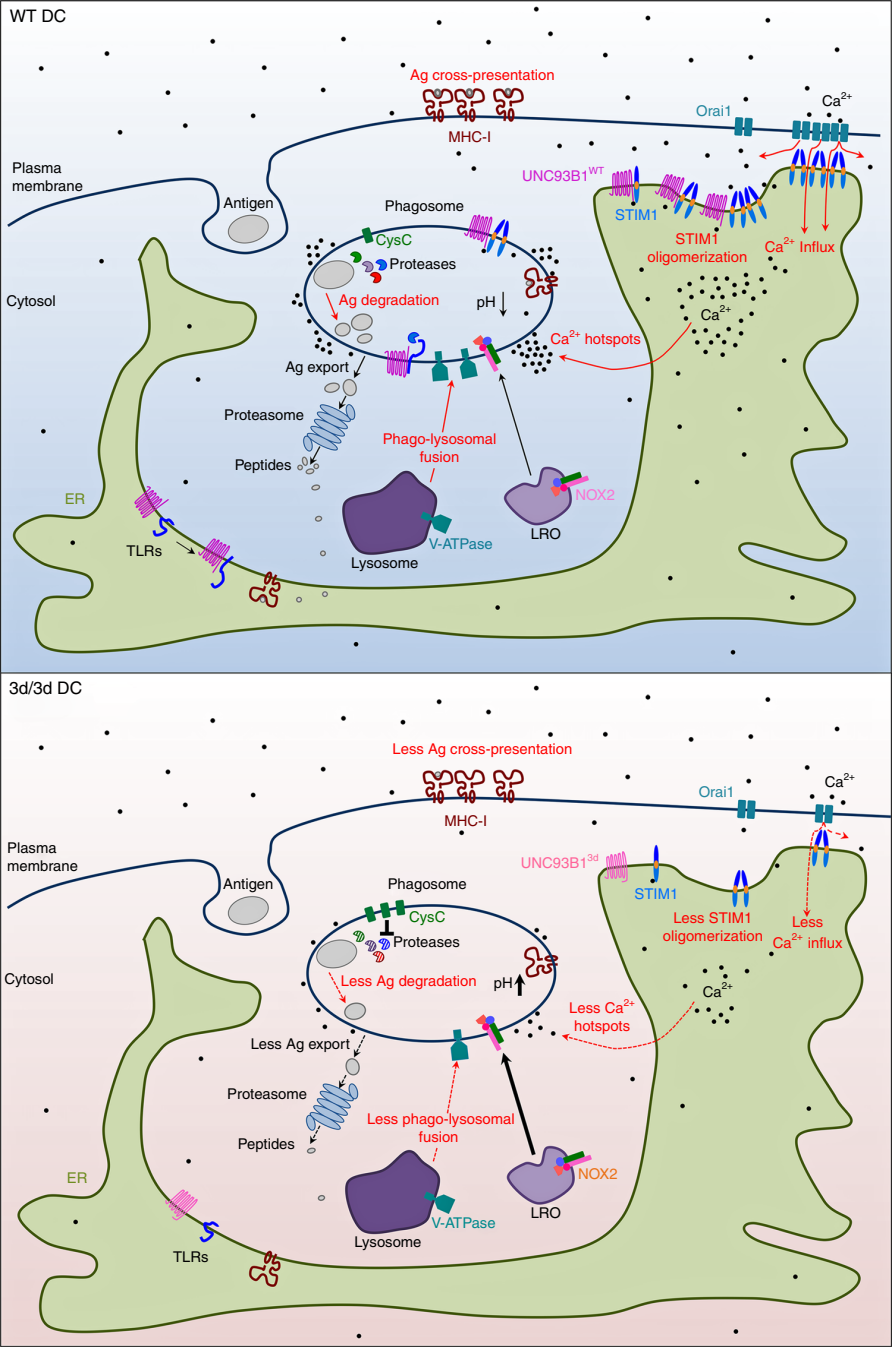
Function of UNC93B1 and STIM1 in Ca^2+^ influx and antigen cross-presentation in DCs. In WT DCs, UNC93B1 associates with STIM1 through their ER-facing domains resulting in STIM1 oligomerization and activation. In DCs carrying the 3d mutation, UNC93B1-STIM1 association is compromised leading to decreased STIM1 oligomerization and activation, and to reduced SOCE and periphagosomal Ca^2+^ signaling. UNC93B1 participates to events critical for efficient cross-presentation either STIM1 dependent (antigen degradation, phago-lysosomal fusion, phagosomal Ca^2+^ hotspots, in red in the scheme) or STIM1 independent (endosomal/phagosomal pH, NOX2 recruitment and ROS production, cathepsins activity and antigen export to the cytosol, TLR folding and transport, highlighted in black). In 3d/3d cells, all these parameters are severely impaired resulting in inefficient antigen cross-presentation. In summary, UNC93B1, together with STIM1, are important regulators of antigen cross-presentation by modulating phagosomal homeostasis and calcium physiology in DCs. Ag: antigen, CysC: cystatin C, LRO: lysosome-related organelle

In summary, our results define UNC93B1 as a novel STIM1 partner and thus regulator of SOCE and calcium homeostasis, giving a new physiological role for UNC93B1 in DCs. Also, we demonstrate that STIM1-dependent Ca^2+^ signaling and UNC93B1 function are critical participants in MHCI antigen cross-presentation (Fig. 8), therefore making them attractive targets for anti-infectious and anti-tumoral therapies.

## Methods

### Mice

C57BL/6 mice were purchased from INSERM U1151 animal facilities and UNC93B1 defective mice (3d/3d) were a gift from Dr B. Ryffel (CDTA, Orléans). Both colonies were bred in our animal facilities and mice were routinely genotyped by PCR. TCR-transgenic mice OT-I (CD45.1^+^) mice were bred in our animal facilities. The experiments were conducted with male or female mice, 8–12-weeks-old, weighing 20–25 g and housed under specific pathogen-free conditions, in our animal facilities at 22 ± 2 °C with a 12 h light dark cycle, with access to food and water ad libitum. All animal care and experimental procedures were performed in accordance with the guidelines and regulations of the French Veterinary Department and approved by ethical committee (code number: A-75–2003).

### Reagents and plasmids

Murine Unc93b1 cDNA construct was cloned into c-flag-pcDNA3.1 or pmCherry-C1 plasmid by PCR of the pUNO mUNC93B1-HA plasmid (Invivogen). The mUnc93b1 construct was mutagenized in its His 412 into Arg using the Quick change mutagenesis kit (Stratagene). STIM1 plasmids (pEX-SP-YFP-STIM1(23-685), pEX-SP-YFP-STIM1(D76A), pEX-SP-YFP-STIM1(1-237), pEX-SP-YFP-STIM1(342-448)) were purchased from Addgene^60^ or were a kind gift from Dr. R. Lewis (Stanford University, USA). The H-2K^b^ expression plasmid was kindly provided by R. Fåhraeus^61^. For STIM1 silencing, the siRNAs were obtained from Qiagen (Cat. no. SI02735068, SI02710736, SI01435630, SI01435623, Product no. Mm_Stim1_6, Mm_Stim1_5, Mm_Stim1_2, Mm_Stim1_1 with the following sequences: AAGAAAGTGATGAGTTCCTAA, CTGGGCAAGGATGTTATATTT, CTGGTTTGCCTATATCCAGAA, CAGCTTTGAGGCCGTCCGAAA, respectively) and siRNA SI02710736 and SI01435623 were chosen. Control negative siRNA was purchased from Qiagen (SI01435623). In some experiments BM-DCs were pre-incubated with ConB (20 nM, Sigma), 0.2 nM concA(concanamycin A) or with 10 μM DPI (Sigma) for 20 min.

### Quantitative PCR

RNA extraction was performed using NucleoSpin RNA (Macherey-Nagel), according to the manufacturer’s protocol. cDNA was obtained with the SuperScriptVILO cDNA synthesis kit (Life Technologies), according to the manufacturer’s protocol, starting from 1 μg of RNA. Quantitative PCR experiments were done with the Lightcycler 480 (Roche) using the Taqman Gene expression assay (Applied Biosystem) with the following primers: Mm01158413_m1 for *Stim1*, Mm01223103_m1 for *Stim2*, and Mm99999915_g1 for *Gapdh* as a control.

### Preparation of primary cells, cell lines, and transfection

Bone marrow-derived DCs (BM-DC) were generated from C57BL/6 and 3d/3d mice by culturing precursors for 8–10 days in GM-CSF-supplemented medium as previously described^30^. 2 × 10^6^ BM-DCs at day 6 were nucleofected with 1.5 μg of cDNA coding for GFP, GFP-tagged STIM1, STIM1-D76A, or with 1 μM siRNA for *Stim1* via the Amaxa kit (Lonza, Germany). 48 or 72 h (for STIM1 silencing) later, cells were harvested and were used different assays. B16-F10-OVA melanoma cell line was kindly provided by Olivier Lantz (Institut Curie, U932, Paris). Cells were maintained at 37 °C in 5% CO_2_ atmosphere in DMEM (10% FBS, 2 mM glutamine, 50 μg/ml penicillin/streptomycin, 50 μM of β-mercaptoethanol, 25 mM HEPES, 1× non-essential amino acids, 1 mM of sodium pyruvate). For inoculation into mice, cells were recovered after 2–3 passages in vitro and resuspended in 150 μl of PBS. Murine fibroblasts were maintained at 37 °C in 5% CO_2_ atmosphere in DMEM (10% FBS, 2 mM glutamine, 50 μg/ml penicillin/streptomycin)^17^ and were transfected with cDNA coding for GFP, GFP-tagged STIM1, STIM1-D76A, STIM1-ΔCT, STIM1-CAD and/or cherry-tagged or FLAG-tagged UNC93B1-WT and UNC93B1-mutated (UNC93B1^3*d*^) with JetPrime Transfection Reagent (Novagen) following the manufacturer’s protocol for adherent cells. HeLa cells were cultured at 37 °C in 5% CO_2_ in MEM (10% FBS, 2 mM glutamine, 50 μg/ml penicillin/streptomycin). Cells were seeded on 25-mm diameter glass coverslips and transfected at 70–80% confluence with 5 μl Lipofectamine 2000 (Invitrogen) by adding 2 μg of plasmid per coverslip and imaged 24 h after transfection. The B3Z hybridoma cell line was kindly provided by N. Shastri (University of California, USA). Cells were maintained at 37 °C in 5% CO_2_ atmosphere in RPMI 1640 (10% FBS, 2 mM glutamine, 50 μg/ml penicillin/streptomycin, 50 μM of β-mercaptoethanol, 25 mM HEPES, 1× non-essential amino acids, 1 mM of sodium pyruvate).

### Microarrays

Data were processed from GSE41496 Gene Expression Omnibus project performed on Agilent-028005 SurePrint G3 Mouse GE 8×60K Microarrays. The original experiments intended to describe the whole transcriptome from spleens of plasmodium Chabaudi infected vs. control mock-infected mice sufficient or deficient for various TLR-related molecules. Among these data, we analyzed control spleens (from UNC93B1 deficient (data sets: GSM1018264, GSM1018265, GSM1018266, GSM1018267) and sufficient (GSM1018240, GSM1018241, GSM1018242, GSM1018243) groups of mice). The description of the set of data can be found here: https://www.ncbi.nlm.nih.gov/geo/query/acc.cgi?acc = GSE41496. Background noise was computed using a custom algorithm within R as follows. Assuming that a maximum of 80% of genes are expressed on any given microarray, we tagged the 20% of probes with the lowest intensity for each microarray as background. A threshold was fixed at two standard deviations over the mean of the background. All probes for which normalized intensities were lower than the computed threshold were designated as background for each array. When comparing gene expression levels between two groups, a probe was included in the analysis if its intensity exceeded the background in at least 80% of the animals from at least 1 group. To identify differentially expressed genes, we used the Student’s *t-*test and lists were filtered at *p* ≤ 5%. Cluster analysis was performed by hierarchical clustering using the Spearman correlation similarity measurement and an average linkage algorithm. Functional analyses were carried out using Ingenuity Pathway Analysis (Qiagen), the R project for Statistical Computing (http://www.r-project.org/), Ingenuity Pathways Analysis (http://www.ingenuity.com), and Gene Expression Omnibus (https://www.ncbi.nlm.nih.gov/geo).

### In vitro and in vivo cross-presentation

In vitro and in vivo cross-presentation assays were performed as previously described^16,17^. Briefly, OVA or BSA-coated beads or splenocytes electroporated with OVA from Balb/C mice (H-2^d^) were incubated with DCs for 6 h. Then, CFSE-labeled OT-I T cells were added to the culture and the proliferation of T cells was monitored 3 days later. DCs incubated with SIINFEKL were used as a proliferation control. For in vivo cross-presentation assays, CFSE-labeled CD8^+^ T cells from OT-I RAG (CD45.1^+^) mice were intravenously injected into CD45.2^+^ recipient mice (WT or 3d/3d), and 1 day later OVAbs or PBS alone were administered intravenously. CFSE dilution of injected T lymphocytes in spleen was analyzed by staining with anti-CD8 and anti-Thy1.1 (OX-7) (BD PharMingen) 72 h later.

### In vivo tumor progression

WT (C57BL/6) or 3d mice were injected subcutaneously with 0.2 × 10^6^ B16-OVA cells (day 0). At day 1, both mice genotypes were injected or not with 2.5 × 10^6^ OT-I T cells (TCR transgenic specific for OVA). The OT-I CD8^+^ T cells used in adoptive transfer were previously enriched from lymph nodes of OT-I mice by depleting cells positive for: CD11c, CD11b, Gr1, CD4, CD19 using immunomagnetic beads (PE Selection Kit, Miltenyi Biotech); the purity of 85% was determined by FACs. Tumor growth, length (1), width (2), and depth (3), was measured with the aid of a caliper every 2–4 days and mice survival was monitored daily. Mice were sacrificed when tumors ulcerated and/or reached the mean diameter of 15 mm. The data obtained were expressed as: mice survival, tumor volume, as determined by using the formula:

1 $${\it{V}} = (4{\rm{/}}3){\kern 1pt} {\rm{pi}}{\kern 1pt} {\rm{r}}1{\kern 1pt} {\rm{r}}2{\kern 1pt} {\rm{r}}3.$$

### Ca^2+^ measurements and imaging

Cytosolic-free calcium concentration ([Ca^2+^]_i_) and SOCE were monitored using a multiplate reader Berthold TriStar^62^ (Berthold France SAS, Thoiry, France). Cells were first plated for 24 h in 24-well plates in BMDCs medium. Cells were loaded with 4 µM fluo4-AM for 45 min and then washed in recording medium containing (mM) NaCl 116, KCl 5.6, MgCl_2_ 1.2, NaH_2_PO_4_ 1, NaHCO_3_ 5, Hepes 20 (pH 7.3) supplemented with 150 µM EGTA. Recordings were performed at 37 °C. TG (1 µM) was added in the absence of external Ca^2+^ and amplitudes of the responses were calculated using Origin software. Total and constitutive calcium entries were determined after the addition of CaCl_2_ (2 mM) in the presence or absence of TG, respectively, and SOCE being determined as the difference between total and constitutive calcium entries. Fluorescence signals were calibrated in individual wells by addition of 25 µM digitonin containing 6 mM Ca^2+^. [Ca^2+^]_i_ was determined using the equation:

2 $$Kd*\left( {\left( {F - Fmin} \right)/\left( {Fmax - F} \right)} \right)$$

with Kd of fluo4 for Ca^2+^ taken as 345 nM, Fmax being the maximum fluorescence signals after digitonin addition, Fmin the minimum fluorescence measured in wells containing the same cell density without the calcium dye.

For live imaging of cell surface Ca^2+^ spots, 15 × 10^4^ BM-DC from WT and 3d/3d mice were seeded on Fluorodishes and were loaded with 4 μM Fura-2-AM, 0.01% pluronic (ThermoFisher) in modified Ringer’s (without Ca^2+^) for 15 min at 37 °C. Cells were subsequently washed before addition of TG (1 μM) at 50 s of recording and CaCl_2_ (2 mM) at 250 s and imaged for up to 500 s. Video images of Ca^2+^-related cell-surface spots were acquired with Inverted Confocal Spinning Disk Andor/Nikon. Quantification of Ca^2+^-related spots per total cell surface were performed with ImageJ.

Ca^2+^ microdomain/hotspot imaging was performed as described^29^ except cells were loaded with 4 μM Fluo-8-AM (AAT Bioquest) for 30 min at 37 °C, 30 min at RT and 2.5 μM BAPTA-AM for the last 10 min, in modified Ringer’s containing 500 μM sulfinpyrazone before exposure to OVAb and hotspots imaged at 30 min, while GSK7975A (a gift from Dr. Martin Lochner, University of Bern, 10 μM) was applied concurrently with OVAb and images taken after 30 min.

### TIRF microscopy

Fibroblasts transfected with STIM1-WT or STIM1-D76A GFP-tagged and UNC93B1-WT or UNC93B1-3d cherry-tagged plasmids were left to adhere on glass slides, stimulated with TG and fixed before visualized by TIRF. TIRF time-lapse videos were recorded for 5 min (exposure time, 200 ms). During the observation, the cells were kept at 37 °C. Fluorescence data were acquired with an Eclipse Ti-E TIRF imaging system (Nikon). Images were acquired with a Roper Scientific QuantEM 512 SC camera (Nikon) and NIS-Elements Advanced Research software (version 3.1). Image sets were processed with ImageJ.

### Duolink, immunoprecipitation, and western blotting

Duolink (OLINK) was performed according to the manufacturer’s instructions and as previously described^17^. Briefly, DCs were grown on coverslips and then fixed in 4% paraformaldehyde for 10 min before permeabilization in PBS/0.05% saponin/0.2% BSA for 10 min. Cells were then blocked in 3% BSA/PBS and primary antibodies were incubated (homemade rabbit anti-UNC93B1 polyclonal antiserum targeting aa 515–598 and affinity purified by Moravia and anti-GFP from Roche for STIM1-WT or STIM-D76A transduced cells). After washing the cells, PLA probes were added, followed by hybridization, ligation, and amplification for 90 min at 37 °C. Nucleus (DAPI labeling) and UNC93B1-STIM1 interactions (red) were visualized after incubation with the detection solution. Slides were analyzed by confocal microscopy. Quantification was performed by using ImageJ software. For western blotting, 5–50 μg of proteins were submitted to separation on a 4–12% SDS Nu-PAGE Bis-Tris gels (Invitrogen). Proteins were transferred on a PVDF membrane and immunodetection was realized. Mouse monoclonal anti-GOK/STIM1 (clone 44/GOK, dilution 1/250, BD Biosciences), rabbit polyclonal anti-STIM1 (AB9870, Millipore), rabbit polyclonal anti-H-2K^b^ (Ab93364, 1/1000, Abcam), mouse monoclonal anti-a-tubulin (clone DM1A, T9026, 1/5000, Sigma), mouse monoclonal anti-b-actin (clone AC-74, A2228, 1/1000, Sigma), rabbit polyclonal anti-FLAG (F7425, 1/500, Sigma), mouse monoclonal anti-GFP (clone 7.1 and 11.3, 1/1000, Roche Diagnostics), rabbit polyclonal anti-V-ATPase (anti-ATP6E sc-20946, 1/500, Santa Cruz), rabbit polyclonal anti-V-ATPase (ab220044, ATP66J, 1/500, Abcam), rabbit polyclonal anti-cystatin-C (ABC20, 1/500, Sigma), mouse monoclonal anti-gp91*phox* (clone53/gp91 *phox*, 611415, 1/500, BD Biosciences), rabbit polyclonal anti-Rab27a (168 003, 1/1000, Synaptic Systems) antibodies were used. For immunoprecipitation of over-expressed proteins in fibroblasts, 4 mg of cell lysate was incubated o/n with protein G beads coated with anti-FLAG antibody or GFP–Trap_MA beads from Chromotek. Beads were then extensively washed and boiled in sample buffer containing 1% SDS. Samples were analyzed by Bis-Tris 4–12% SDS-NuPAGE and developed with HRP-coupled substrate (Amersham). For immunoprecipitation of endogenous STIM1–UNC93B1 complexes in BM-DC, cells were lysed in buffer containing 50 mM Tris-HCl, pH 7.4, 150 mM NaCl, and 5 mM EDTA with 1% NP-40 as detergent and protease inhibitors. Lysates were immunoprecipitated with protein G beads coated with anti-STIM1 antibody (rabbit monoclonal anti-STIM1 ab108994 from Abcam). Washes were performed with 0.1–0.5% NP-40 containing lysis buffer. Immunoprecipitates were dissolved by denaturation with 1% SDS and 2% β-mercaptoethanoland subjected to 4–12% SDS–PAGE with boiling the samples for STIM1 immunodetection or without heating the samples for UNC93B1 visualization (homemade rabbit anti-UNC93B1).

### FRET measurements

STIM1 oligomerization was followed in HeLa cells in the presence of either Cherry-tagged WT or 3d-mutated UNC93B1 by measuring the increase in the apparent FRET efficiency of YFP-tagged and CFP-tagged STIM1 upon stimulation with 1 μM TG in modified Ringer’s buffer^29^. FRET measurements were performed using the E_app_ E-FRET method of Zal and Gascoigne^63^, where:

3 $${\rm{FRET}}\,{\rm{efficiency}} = \left( {{I_{DA}}-a{I_{AA}} - d{I_{DD}}} \right)/({I_{DA}}-a{I_{AA}} + \left( {G - d} \right){I_{DD}}).$$

Briefly, time-lapse images were acquired every 12 s in three channels using the following excitation/emission (in nm) configurations: CFP donor intensity (*I*_DD_): 430/510; YFP acceptor intensity (*I*_AA_): 488/530; YFP FRET-induced acceptor intensity (*I*_DA_): 430/530. The bleed-through coefficients *a* and *d* were estimated by acquiring images of cells transfected with only YFP-STIM1 or only CFP-STIM1, and measuring the slope of the linear regression of *I*_AA_ vs. *I*_DA_ or *I*_DD_ vs. *I*_DA_ plotted values, respectively, for 80–90 cells of varying expression levels. The G parameter was estimated by completely photobleaching YFP-STIM1 in cells co-expressing CFP-STIM1, through continuous 488 nm light exposure for 40 min, and acquiring pre and post-photobleaching 3-channel fluorescence using the configuration above. Thus, *G* is calculated as:

*G* = ((*I*_DA_−*aI*_AA_−d*I*_DD_)−(*I* ^post^_DA_−*aI* ^post^_AA_−d*I* ^post^_DD_))/(*I* ^post^_DA_−*I*_DD_) [4]

Images were acquired for 2 min before TG addition and for 10 min after, and single-cell fluorescence measurements extracted with ImageJ. Traces were normalized to the average FRET efficiency of the last minute before TG addition, smoothed using a 2nd order 4-neighbor smoothing, and fitted to a Boltzman sigmoidal curve using GraphPad Prism software. The V50 parameter of the curve fit is reported as the time-to-half–maximum FRET efficiency, and the TOP parameter as the maximum FRET efficiency.

### Phagocytosis

For flow cytometry-based phagocytosis assays, 3 µm NH2 beads (Polysciences, Inc.) were activated with 2 mg/ml sulfo-NHS-LC-Biotin, washed with PBS—glycine 1 M and labeled with streptavidin A488 before added at different ratios to the BM-DC for 30 min at 37 °C. Cells were washed and resuspended in Trypan Blue 0.2 mg/ml/1 M citrate pH 4.0 buffer immediately before FACS analysis. The percentage of fluorescence represents only the particles that are internalized and are not bound in the cell surface. Treatment of cells with 10 μM of Cytochalasin D (Sigma) prior to beads administration was used as a control for phagocytosis.

### Endosomal and phagosomal pH

Endosomal pH was measured as previously described^16^. Briefly, cells were pulsed with 1 mg/ml of FITC-labeled and Alexa-647-labeled 40 kDa dextrans (Molecular Probes) for 10 min at 37 °C, extensively washed with cold PBS plus 1% BSA then chased for 50 min and analyzed by FACS, via a FL1/FL4 gate selective for cells that have endocytosed the fluorescent probes. Phagosomal pH was measured using FITC-coupled zymosan added at 20:1 ratio as described^28^ except that calibrations (pH 4.0–pH 9.0) were performed using five different stacks per condition for each calibration buffer rather than a single time-lapse acquisition. FITC-zymosan was incubated in 20 mg/ml OVA overnight and washed 3× in PBS. OVA-opsonized zymosan were added to cells grown on Fluorodishes in complete medium and were washed in modified Ringer’s and imaged 90 min after addition. Cells were outlined in white and the images were a merge of the brightfield and 480/440 color-coded ratio channels 90 min after addition of FITC-OVA-zymosan. pH values were obtained from calibration curves matched to the ratio color-coded bar.

### Phagosomal OVA degradation assay and phagosome preparation

100 × 10^6^ BM-DCs were pulsed for 20 min at 37 °C with magnetic particles (Invitrogen) and then chased at 37 °C for 100 min. Cells were then washed with PBS and mechanically disrupted by passing them through 25 g needle. Phagosomes were purified by magnetic separation, washed, and lysed. Equal amounts of proteins were submitted to separation on a 4–12% SDS NuPAGE Bis-Tris gels (Invitrogen). Proteins were transferred on a PVDF membrane and immunodetection was realized with anti-gp91*phox*, anti-V-ATPase, anti-cystatin C, or anti-Rab27a antibodies. Phagosomal OVA degradation assay was performed as previously described^16^. Briefly, 3 μm NH2-latex beads (Polysciences, Inc.) were covalently coated with OVA (0.2 mg/ml, OVA, Low Endo, Purified from Worthington Biochemical Corporation) according to manufacturer’s instructions. BM-DCs were pulsed for 15 min at 37 °C and chased for different times at 37 °C. Cells were lysed in 50 mM Tris-HCl (pH 7.4) supplemented with 150 mM NaCl, 1 mM DTT, 0.5% NP-40, and cocktail of protease inhibitors (Roche). For FACS analysis, beads were stained with rabbit polyclonal anti-OVA (SAB4301164, 1/100, Sigma) and a secondary goat anti-rabbit Alexa488 (A-11034, 1/200, Molecular Probes).

### Phago-lysosome fusion assay

Phago-lysosome fusion was measured using a protocol modified from Podinovskaia et al.^64^. 3.0 μm polybead Amino Microspheres (Polysciences) were labeled with Alexa-488-SE and coated with OVA by washing beads in 25 mM sodium citrate, 25 mM sodium phosphate buffer, pH 5.0, and incubating beads with 20 mg/ml endotoxin-free chicken OVA (InvivoGen, USA) overnight at 4 ^o^C on an end-over-end rotor, followed by three washes in sterile PBS (ThermoFisher). 24 h prior to the assay cells were pulse labeled with 20 μg/ml Alexa-594-HA (ThermoFisher) in complete medium for 3 h, washed, and followed by a chase period of 24 h to allow the dye to accumulate in lysosomes. Alexa488-OVA beads were added to cells at 20:1 in complete medium, were washed in modified Ringer’s after 30 min, and imaged using alternate 555/590, 488/530, 488/590 nm Ex/Em and brightfield illumination 30 and/or 90 min after addition. When applicable, 0.2 nM ConcA was added just prior to beads. Image analysis was performed using ImageJ software (NIH) on maximum projections of at least 5 × 15 μm *z*-stacks per condition. The P-L fusion index is computed by obtaining the fluorescence signal for the FRET (488/590) and Alexa-488 bead (488/530) channels using semi-automated segmentation based on a single visually determined threshold for the bead channel. FRET ratios (FRET/bead channel) minus the basal (external bead) FRET ratio were normalized to the total average cellular Alexa-594-HA fluorescence obtained from sum projections of the Alexa-594 (550/590) channel and multiplied by 10,000 to obtain the final P-L fusion index.

### ROS measurements

Intracellular ROS were measured on cells on cells seeded at 50,000 cells/well in black/clear bottom plates. 30 μM dihydroethidium was loaded for 1 h in complete medium at 37 ^o^C before washing in modified Ringer’s. Fluorescence was measured every 2 min for 90 min at 37 ^o^C after addition of 20:1 OVAb using a SpectraMAX Paradigm (Molecular Devices) plate reader, and is reported as the maximum fluorescence value normalized to the average baseline value over the first 6 min. Intraphagosomal ROS was measured on cells plated on Fluorodishes using OxyBurst/Alexa-568-coupled zymosan opsonized with OVA. 20 mg of boiled zymosan were washed 3× in sterile filtered 0.1 M NaCO_3_ buffer (pH 8.0), 20 μg/ml OxyBurst-H2DCFDA-SE (ThermoFisher) was added and particles were agitated overnight at RT in tubes filled with nitrogen. Alexa-568-SE (ThermoFisher) was then added for 1 h. Unbound dye was removed by washing in 50, 33, then 25% DMSO/PBS prior to opsonization and zymosan were stored under nitrogen. OxyBurst/Alexa568-OVA zymosan were added to cells at 20:1 in complete medium, were washed in modified Ringer’s after 30 min to remove uningested zymosan, and imaged using alternate 555/590, 488/530 nm Ex/Em and brightfield illumination 30 and/or 90 min after addition. Where applicable cells were pre-incubated for 10 min with 10 μM DPI prior to stimulus addition. Cell borders were delineated using the brightfield image and phagosomes were defined by the Alexa-568 fluorescence within cell borders, using ImageJ software (NIH). The ratio of phagosomal OxyBurst to phagosomal Alexa-568 fluorescence minus the basal (external zymosan) ratio was calculated as an index of intracellular/phagosomal ROS.

### Measurement of antigen export to the cytosol

Measurement of antigen export to the cytosol was performed as previously described^32^. Briefly, cells were loaded with 4 μM CCF4-probe (a FRET-sensitive cytosolic substrate of β-lactamase purchased from Life Technologies) for 1 h, were subsequently washed with PBS and incubated at 37 °C with 2 mg/mL β-lactamase (Sigma) for different time points. Reactions were stopped with cold PBS and live, single, CD11c^+^MHCII^+^ cells were analyzed by flow cytometry by monitoring the increase in 450 nm fluorescence resulting from CCF4 cleavage and loss of 535 nm emission fluorescence.

### Protease activity assay

Protease activity assays were performed on Mithras LB 940 microplate reader by measuring the release of fluorescent N-Acetyl-Methyl-Coumarin in citrate buffer (pH 5.5) at 37 °C. Specific substrates for AEP (Z-Ala-Ala-Asn-NHMec), CatB/L (Z-Phe-Arg-NHMec), and CatS (Z-Val-Val-Arg-NHMec) were from Bachem.

### Flow cytometry

Cells were blocked with Fc block (clone 2.4G2; BD Biosciences) and stained with anti-H-2K^b^ biotinylated (clone KH95; BD Biosciences), anti-MHCII-IA/IE A647 (clone M5/114.15.2; BioLegend) or A700 (clone 56-5321-82; eBioscience), anti-CD80 PE (clone 16-10A1; BD Biosciences), and anti-CD11c BV421 (clone N418; BioLegend) or BV711 (clone 563048; BD-Biosciences). Stained cells were acquired on a BD LSR Fortessa cell analyzer and analyzed with FlowJo (Treestar) software.

### Statistical analysis

Statistical significance was determined by unpaired/paired *t*-test or two-way ANOVA or by Log-Rank (survival curves) using GraphPad Prism 6 software. *P < 0.05, **P < 0.01, ***P < 0.001, and ****P < 0.0001.

### Data availability

Data referenced in this study are available in GEO with the accession code GSE41496. Other data that support the findings of this study are available from the corresponding author upon request.

## Electronic supplementary material

Supplementary information
