# Supplementary material for: UNC93B1 interacts with the calcium sensor STIM1 for efficient antigen cross-presentation in dendritic cells
Source: Nat Commun. 2017 Nov 21;8:1640. doi: 10.1038/s41467-017-01601-5 (PMC5696382; doi:10.1038/s41467-017-01601-5)

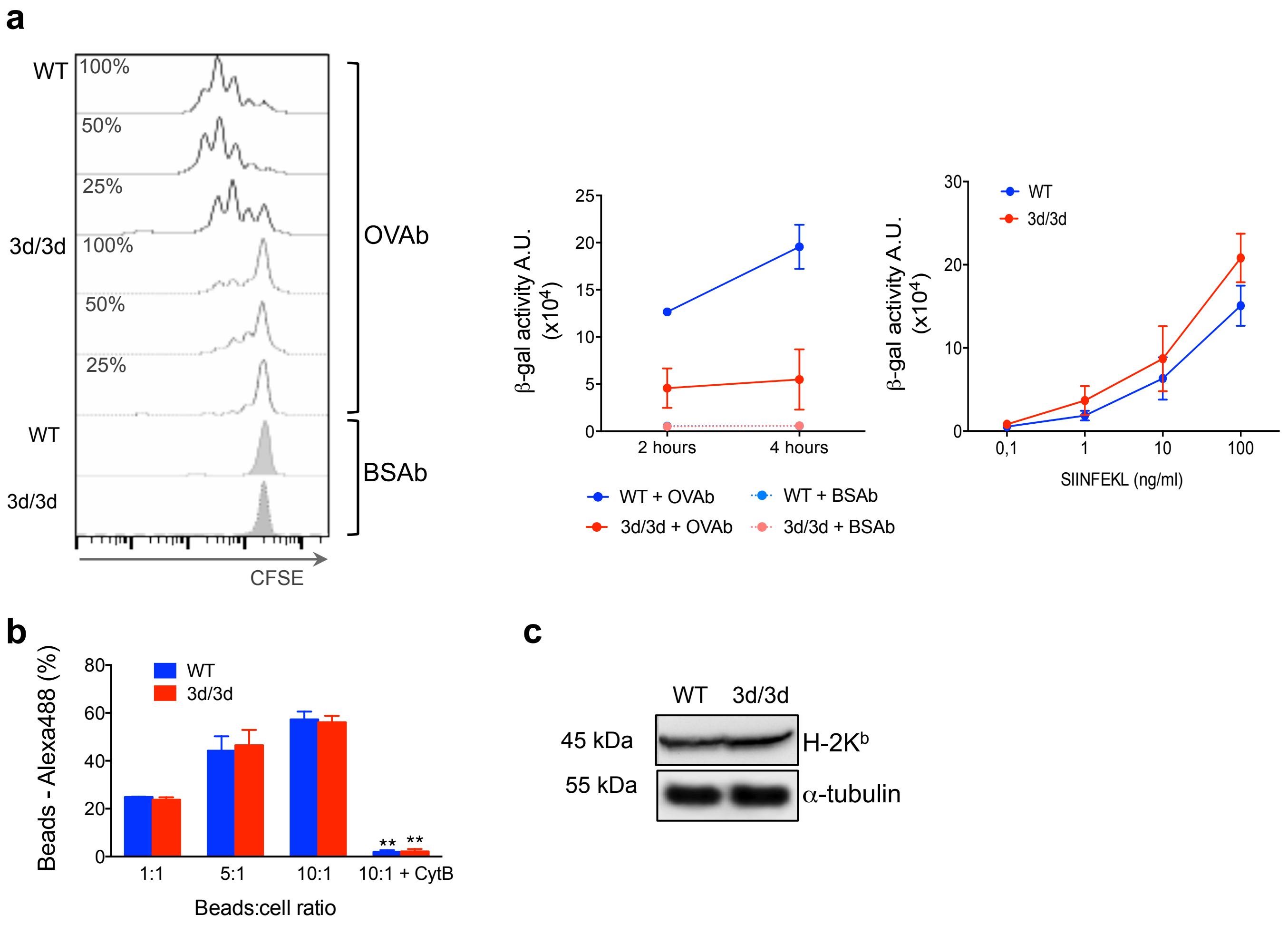

Supplementary Figure 1 | UNC93B1 regulates antigen cross-presentation. WT and 3d/3d BM-DCs were stimulated with different concentrations of OVAb or SIINFEKL peptide for 2, 4 or 6 hours before co-cultured with CFSE-labelled OT-I T cells or B3Z hybridoma for 72 hours or 16 hours respectively. T cell activation was monitored by CFSE dilution for OT-I T cell proliferation or by measuring β-galactodisade activity for B3Z hybridoma. Graphs show mean ± S.E.M. (n=3) and histograms of CFSE dilution are representative from one experiment out of three. (**b**) WT and 3d/3d BM-DCs were left to phagocytose streptavidin A488-beads (3µm) for 30 minutes at 37°C at different ratios and phagocytic capacity was assessed by flow cytometry. Treatment of cells with cytochalasin D (CytB) was performed as a negative control of phagocytosis. Graph shows mean ± S.E.M. (n=2) via unpaired *t* test ** $P < 0.01$. (**c**) Immunodetection of MHCI (H-$2K^b$) and tubulin proteins in WT and 3d/3d BM-DCs lysate. Data are representative of two experiments.

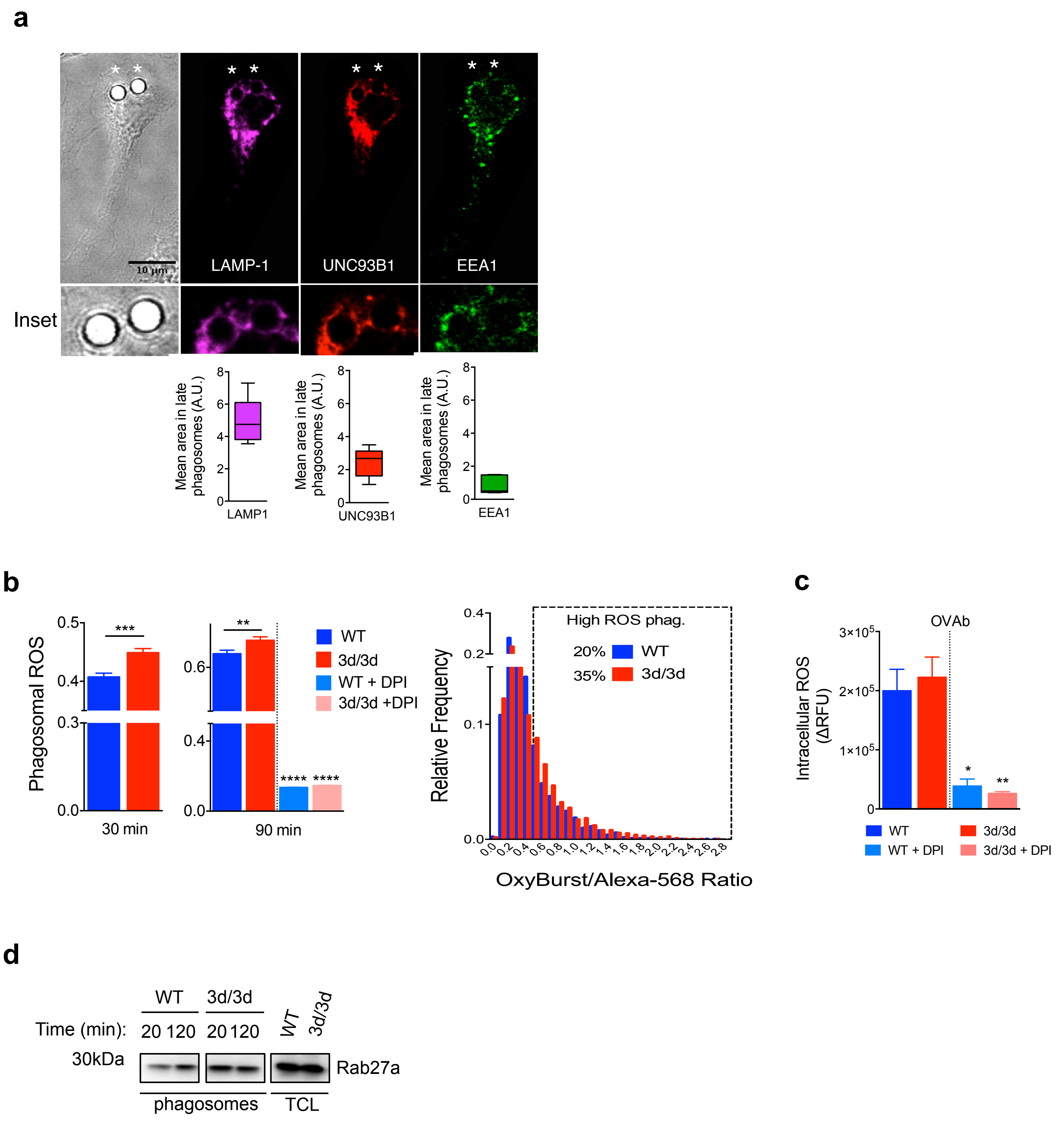

Supplementary Figure 2 Maschalidi et al.

Supplementary Figure 2 | Phagosomal ROS production and maturation are altered in 3d/3d DC (**a**) IF detection of LAMP1 (magenta), UNC93B1 (red), EEA1 (green) in BM-DC after 2 hours phagocytosis of 3µm beads. Asterisks indicate internalised beads (phagosomes). Quantification of mean area of indicated proteins in late phagosomes (*n* = 15 cells). Bars = 10 µm. (**b**) Phagosomal ROS production was monitored by exposing DC to zymosan coupled to OxyBurst and Alexa-568 in the absence or presence of DPI (10 μM). (n=4/5/5 (coverslips each) containing 2709/3275/3663 (WT) or 2882/3907/2957 (3d/3d) phagosomes for 30 min/90 min/DPI; $^{**}P < 0.01$; $^{***}P < 0.001$; $^{****}P < 0.0001$ via unpaired *t* test. Relative frequency of high phagosomal ROS in WT and 3d DC assessed as in left panel. (**c**) Intracellular ROS production was measured in immature DC loaded with 30 µM DHE and exposed to either OVA-coated beads (OVAb) alone or in the presence of DPI. Graph shows mean ± S.E.M. from 4 independent experiments in triplicate wells via unpaired *t* test * $P < 0.05$; ** $P < 0.01$. (**d**) Phagosomes from WT or 3d/3d DC were magnetically purified after 20 min or 2 hours of cargo internalization. Protein expression of Rab27a was visualized by immunoblot either in total lysate (TCL, 50 µg) or in phagosomes (20 µg). Data are representative of two experiments.

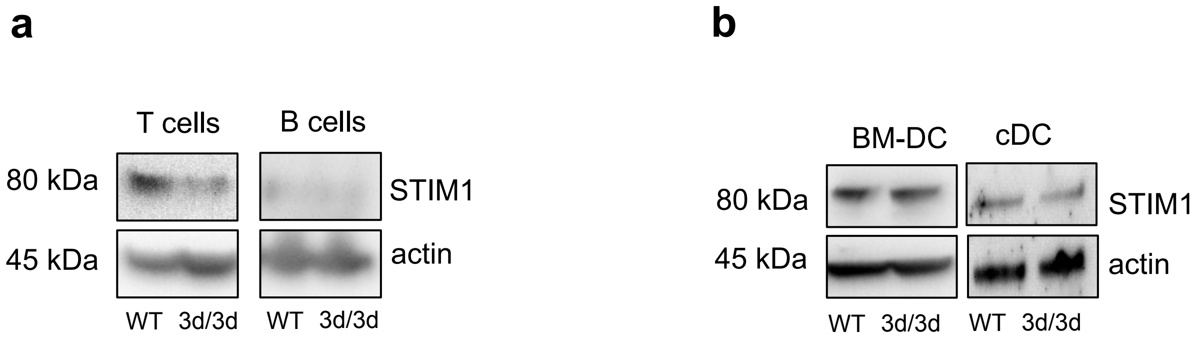

Supplementary Figure 3 | Expression of STIM1 protein is downregulated in 3d/3d T cells but not in DC. Immunodetection of STIM1 and actin (as a loading control) in lysates from (**a**) T and B cells and (**b**) BM-DC or spleen conventional dendritic cells (cDC) from WT and 3d/3d mice.

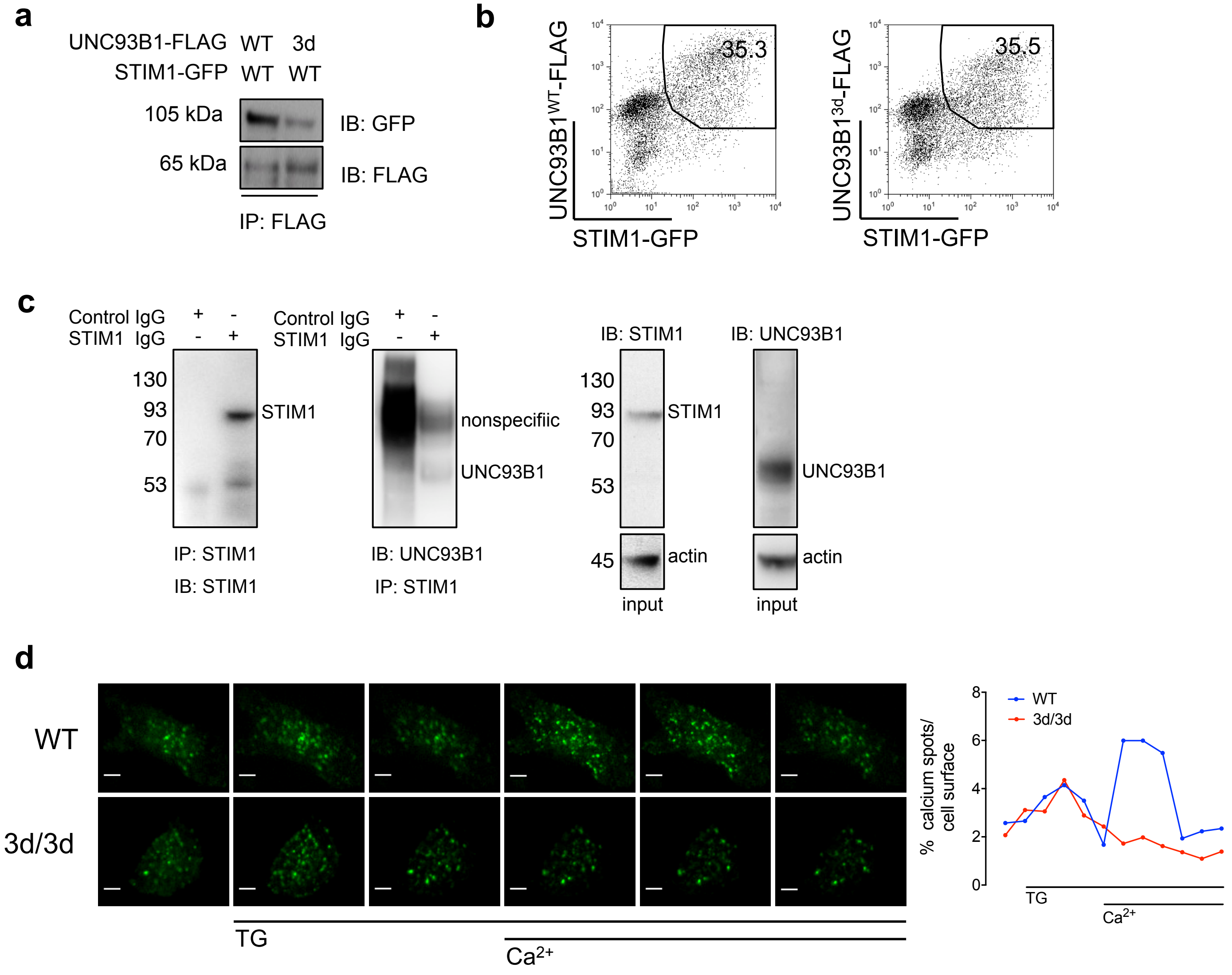

Supplementary Figure 4 Maschalidi et al.

Supplementary Figure 4 | STIM1 interacts with UNC93B1 and its function is impaired in 3d/3d cells. (**a**) Fibroblasts were transfected with STIM1-WT (GFP-tagged) and WT- or 3d mutated UNC93B1-FLAG-tagged plasmids and co-immunoprecipitated with anti-FLAG antibody (UNC93B1) followed by immunoblotting with anti-GFP and anti-FLAG antibodies. One representative immunoblot analysis of three independent experiments is shown. (**b**) Percentage of transfection efficiency of fibroblasts transfected with STIM1-WT-GFP tagged and WT- or 3d mutated UNC93B1-FLAG tagged plasmids assessed by flow cytometry. Graph shows representative dot plots from one out of three independent experiments. (**c**) For Endogenous STIM1-UNC93B1 complexes in DCs were lysed and lysate was immunoprecipitated with protein G beads coated with anti-STIM1 antibody. Immunoprecipitates were thoroughly washed, dissolved by denaturation with 1% SDS and 2% β-mercaptoethanol and subjected to SDS–PAGE with boiling the samples for STIM1 immunodetection (left) or without heating the samples for UNC93B1 visualization (middle). Immunoprecipitation controls for STIM1, UNC93B1 and actin detection in input lysate are shown (right). One representative immunoblot analysis of two independent experiments is shown. (**d**) Representative live imaging of cell surface Ca^{2+} spots in WT or 3d/3d DC loaded with Fura-2-AM and pluronic (see Methods section) treated with thapsigargin (TG) at 50 sec of recording in Ca^{2+}-free Ringer's solution followed by stimulation with Ringer's solution supplemented with 2 mM Ca^{2+} at 250 sec and imaged for up to 550 sec (left). Bars = 10 μm. Quantification of the percentage of Ca^{2+}-related spots per total cell surface indicate impaired Ca^{2+} influx in 3d/3d DC (right).

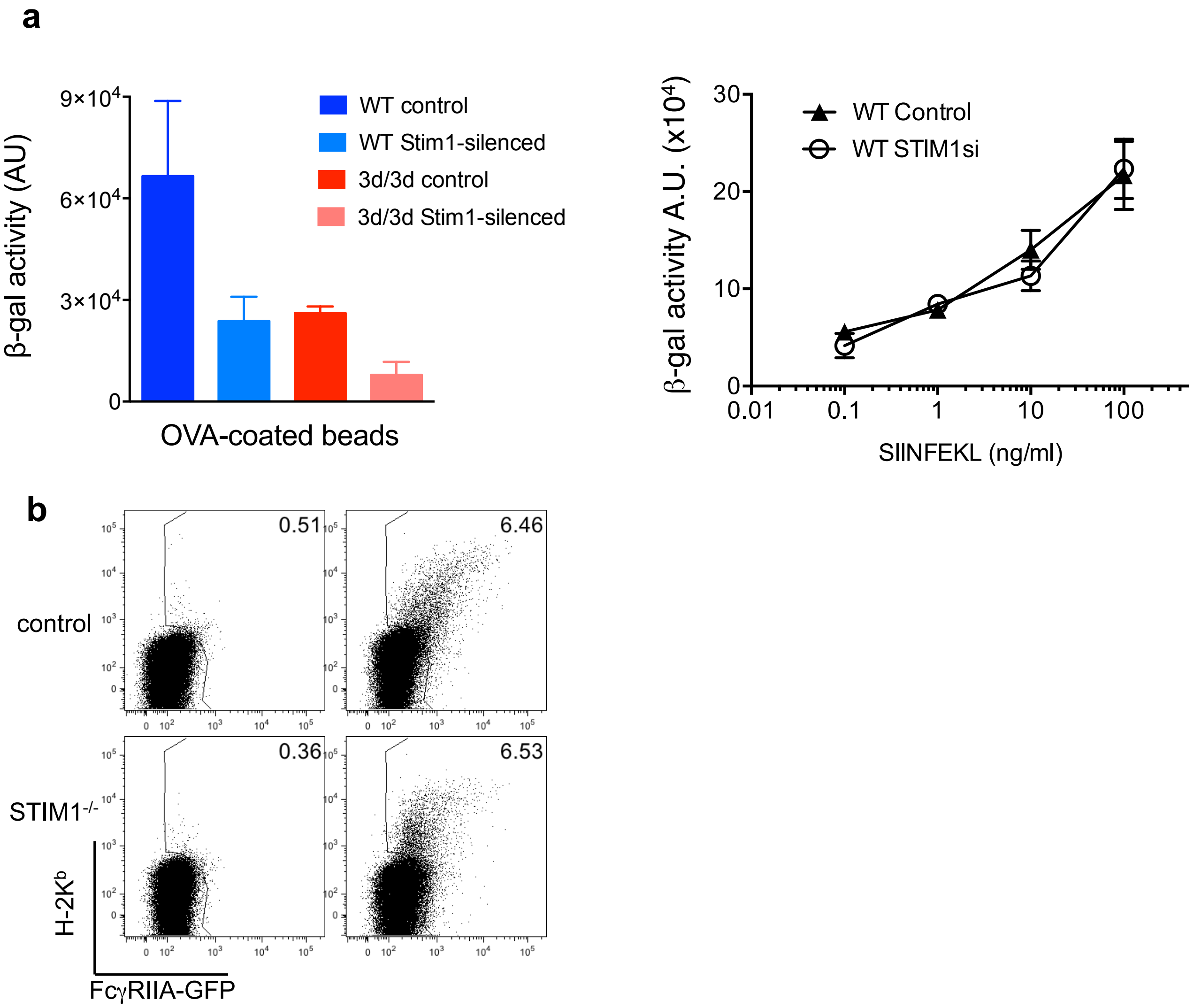

Supplementary Figure 5 | Silencing STIM1 in DC inhibits antigen cross presentation (**a**) WT and 3d/3d DC silenced or not for STIM1 were stimulated with OVAb (left panel) or SIINFEKL peptide (right panel) for 2 hours before co-cultured with B3Z hybridoma for 16 hours. T cell activation was monitored by measuring β-galactosidase activity. Graphs show mean ± S.E.M. (n=3) (**b**) Percentage of transfection efficiency of human fibroblasts from control and STIM1-deficient patient co-transfected with FcγRIIA-GFP and H-2K^{b} plasmids (right panel) compared to non-transfected fibroblasts (left panel) as assessed by flow cytometry. Graph shows representative dot plots from one out of three independent experiments.

a

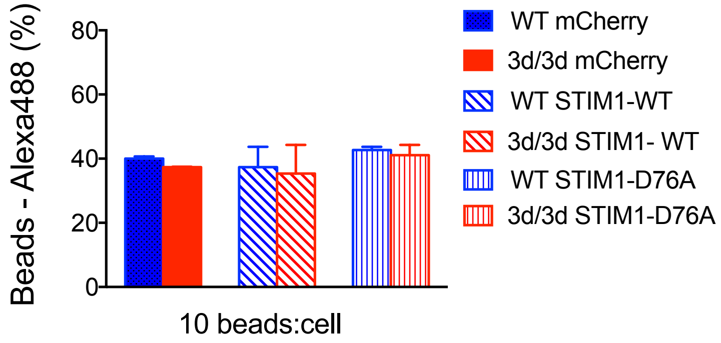

b

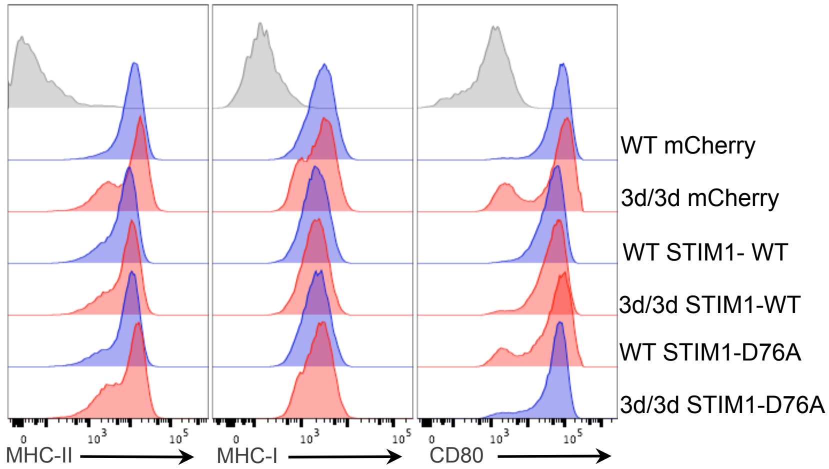

Supplementary Figure 6 | STIM1 active form does not alter DCs phagocytosis or activation. (**a**) WT and 3d/3d BM-DCs transfected with STIM1-WT or STIM1-D76A were left to phagocytose streptavidin A488-beads (3µm) for 30 minutes at 37°C at different ratios and phagocytic capacity was assessed by flow cytometry. Graph shows mean ± S.E.M. (n=2). (**b**) MHCII, MHCI and CD80 surface expression were assessed in WT and 3d/3d BM-DC transfected with STIM1-WT or STIM1-D76A by flow cytometry. Graphs are representative of two experiments.

Figure 2d

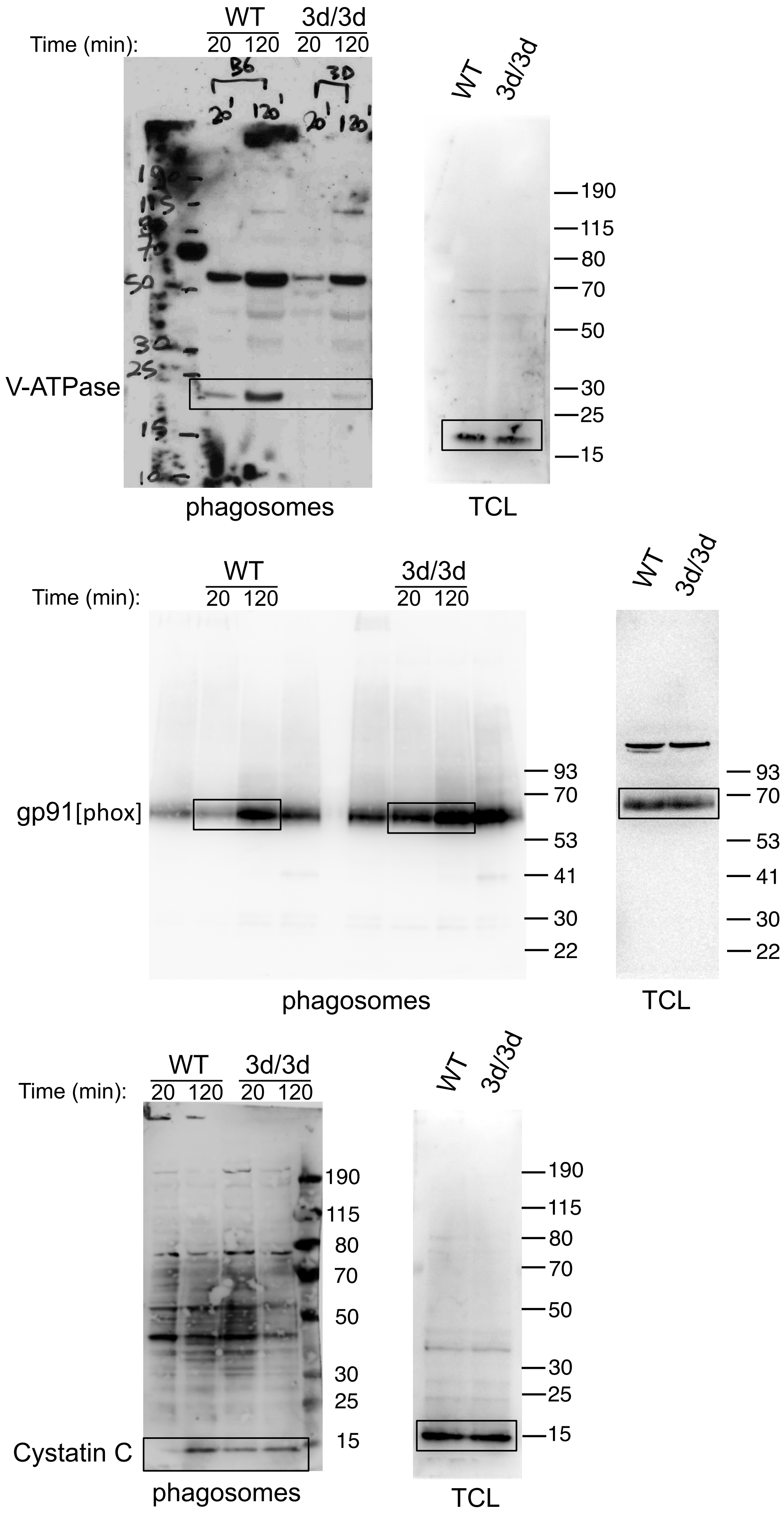
WT
3d/3d
Time (min):
20 120 20 120
V-ATPase
phagosomes
WT
3d/3d
190
115
80
70
50
30
25
15
TCL
WT
3d/3d
Time (min):
20 120
20 120
gp91[phox]
93
70
53
41
30
22
phagosomes
WT
3d/3d
93
70
53
41
30
22
TCL
WT
3d/3d
Time (min):
20 120 20 120
Cystatin C
190
115
80
70
50
30
25
15
phagosomes
WT
3d/3d
190
115
80
70
50
30
25
15
TCL

Figure 4a

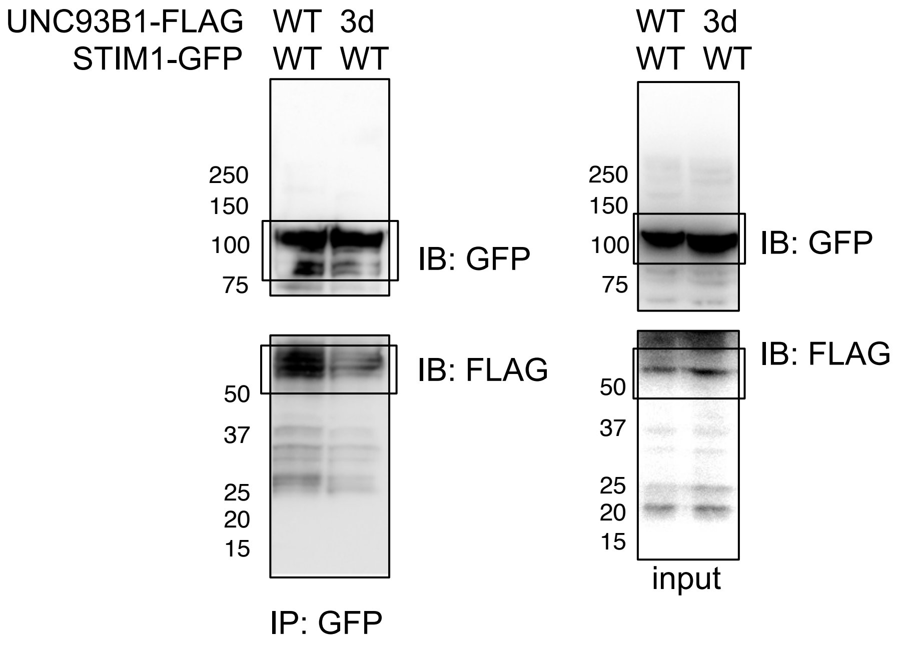

Figure 5b

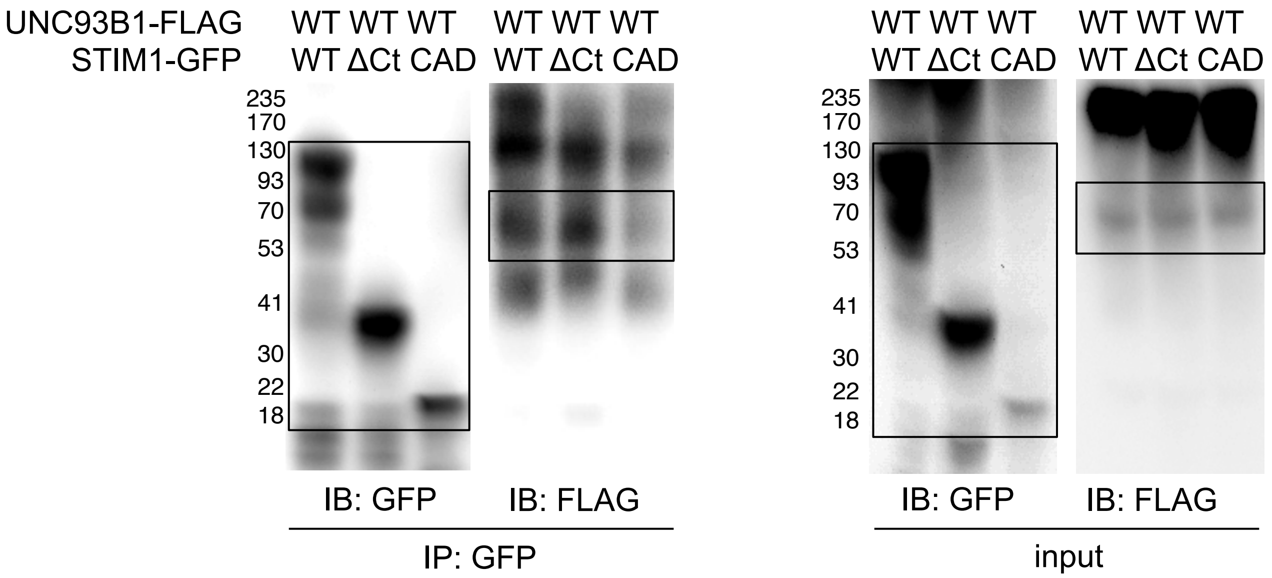

Figure 6b

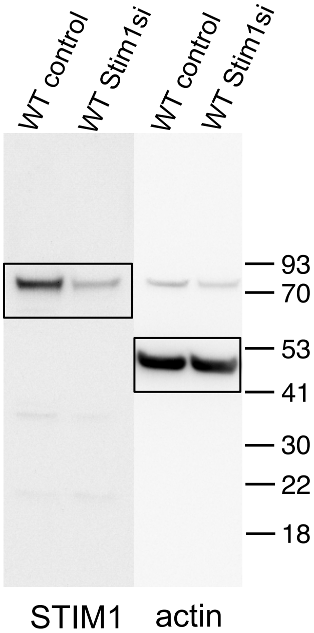

Figure 6g

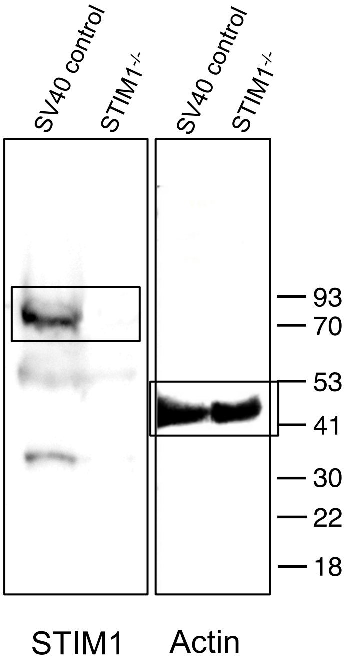

Figure 7

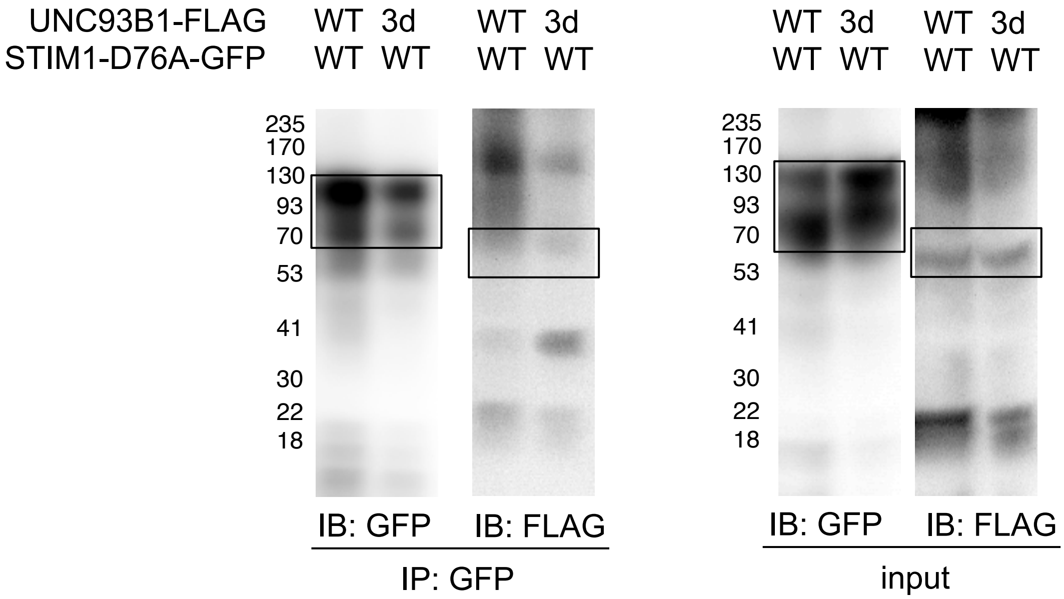

Supplementary Figure 1

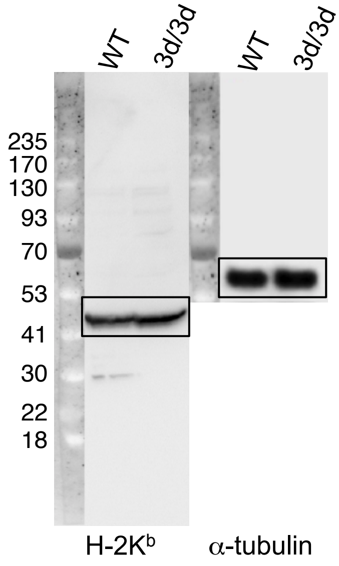

Supplementary Figure 2d

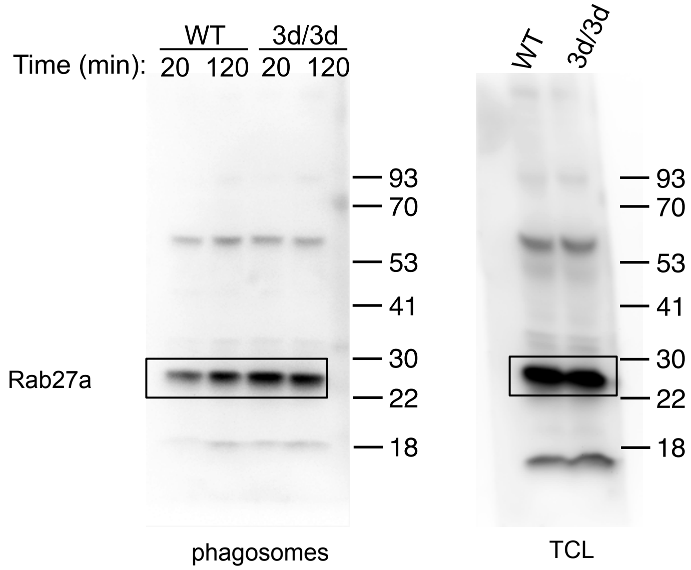

Supplementary Figure 3a

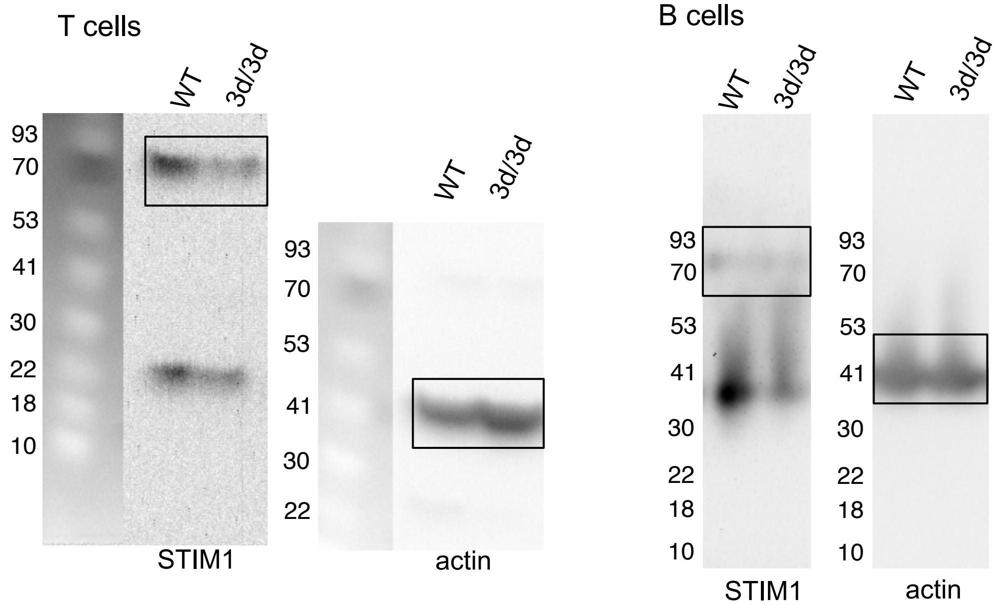

Supplementary Figure 3b

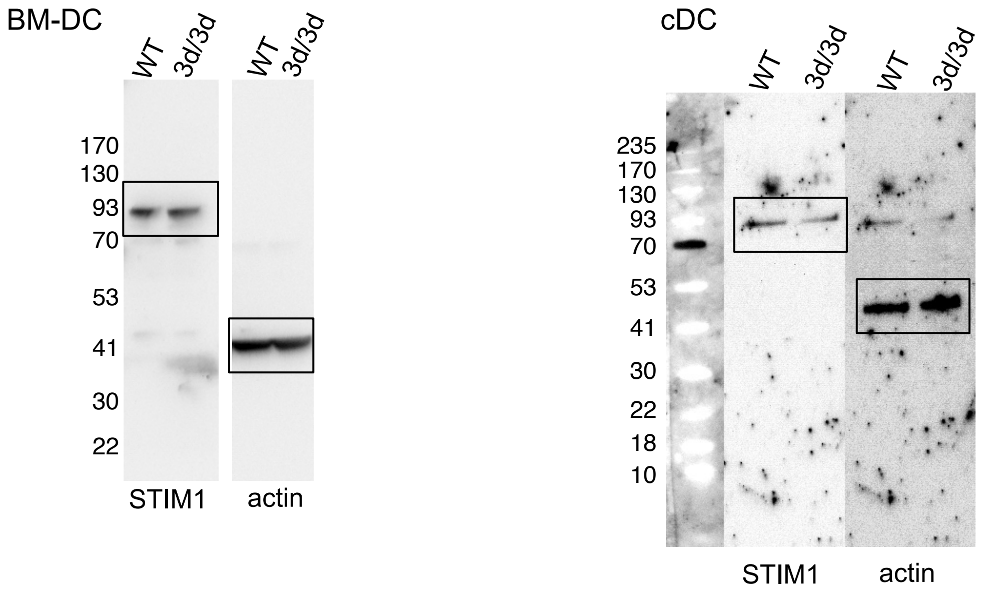

Supplementary Figure 4a

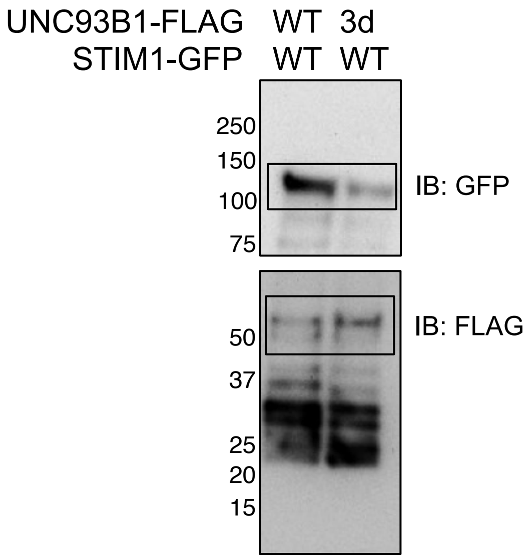

Supplementary Figure 4c

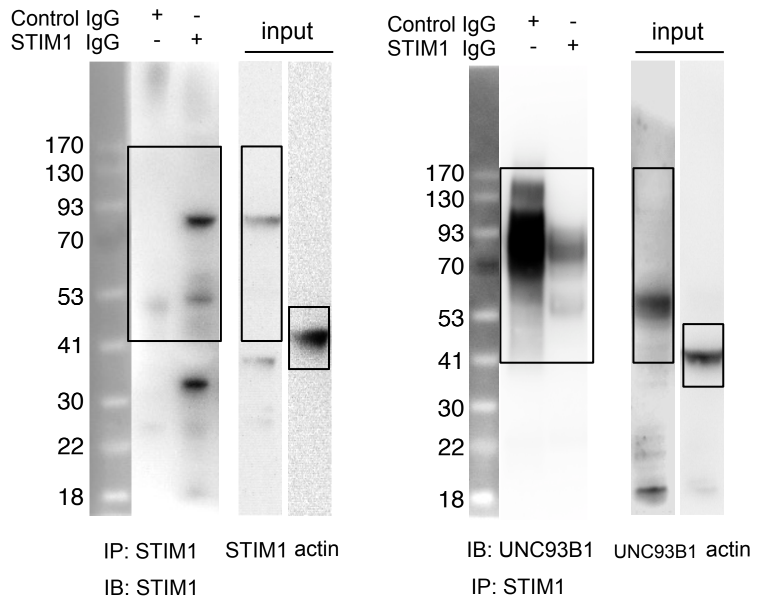

Supplement: Supplementary file 1 — Supplementary information [file 41467_2017_1601_MOESM1_ESM.pdf]
